# Topology-engineered metallic glass metamaterial-supported NiMo nanoglass catalysts for ultra-efficient water splitting

**DOI:** 10.1093/nsr/nwag442

**Published:** 2026-07-17

**Authors:** Congrui Yang, Chaoqun Pei, Haibo Ke, Shilong Sun, Huaping Zhang, Di Ouyang, Lei Zhang, Huaping Ding, Yongyun Zhang, Yuyang Qian, Baoan Sun, K C Chan, Wei-Hua Wang

**Affiliations:** Department of Frontier Research, Songshan Lake Materials Laboratory, Dongguan 523808, China; Research Institute for Advanced Manufacturing, Department of Industrial and Systems Engineering, The Hong Kong Polytechnic University, Hong Kong, China; Department of Frontier Research, Songshan Lake Materials Laboratory, Dongguan 523808, China; Shenzhen Key Laboratory of High Performance Nontraditional Manufacturing, College of Mechatronics and Control Engineering, Shenzhen University, Shenzhen 518060, China; Department of Frontier Research, Songshan Lake Materials Laboratory, Dongguan 523808, China; Dongguan Institute of Materials Science and Technology, Chinese Academy of Sciences, Dongguan 523808, China; Department of Frontier Research, Songshan Lake Materials Laboratory, Dongguan 523808, China; Department of Frontier Research, Songshan Lake Materials Laboratory, Dongguan 523808, China; Dongguan Institute of Materials Science and Technology, Chinese Academy of Sciences, Dongguan 523808, China; Department of Metallic Materials Engineering, College of Materials Science and Engineering, Wuhan University of Technology, Wuhan 430074, China; Department of Mechanical Engineering, City University of Hong Kong, Hong Kong, China; Department of Frontier Research, Songshan Lake Materials Laboratory, Dongguan 523808, China; Department of Frontier Research, Songshan Lake Materials Laboratory, Dongguan 523808, China; Department of Frontier Research, Songshan Lake Materials Laboratory, Dongguan 523808, China; Department of Frontier Research, Songshan Lake Materials Laboratory, Dongguan 523808, China; Research Institute for Advanced Manufacturing, Department of Industrial and Systems Engineering, The Hong Kong Polytechnic University, Hong Kong, China; Department of Frontier Research, Songshan Lake Materials Laboratory, Dongguan 523808, China; Dongguan Institute of Materials Science and Technology, Chinese Academy of Sciences, Dongguan 523808, China

**Keywords:** additive manufacturing, metamaterial catalysts, overall water splitting, superhydrophilic/superaerophobic surface, heterostructure

## Abstract

Electrocatalytic water splitting represents a cornerstone for sustainable hydrogen production, yet its industrial implementation remains constrained by inherent trade-offs among activity, stability and mass transport efficiency. Achieving concurrent optimization of these properties in an integrated catalyst is challenging, as they are dictated by structures spanning multiple length scales. Here, we report a multiscale metamaterial catalyst design strategy that couples topology-engineered transport pathways with nanoglass heterointerface-regulated catalytic activity. Specifically, multiscale metamaterial catalysts (i.e. NiMo@Zr MMCs) are constructed by integrating micro-laser powder bed fusion-fabricated Zr-based metallic-glass triply periodic minimal surface scaffolds with pulsed-electrodeposition (PED)-derived NiMo nanoglass heterostructures. The Gyroid architecture provides enhanced permeability, efficient electrolyte/gas transport and mechanical robustness, while the abundant amorphous–amorphous interfaces within the NiMo nanoglass heterostructure optimize the local electronic environment and catalytic activity. Through the synergistic integration of topology engineering, metallic-glass scaffolds and nanoglass interface engineering, the resulting NiMo@G catalyst delivers record-low cell voltages for overall water splitting (1.438 V at 100 mA cm^−2^ in 1 M KOH) and outstanding long-term durability (>200 h). This work extends the concept of metamaterial catalysts from topology-controlled transport design to the coupling of transport and catalytic-interface engineering across multiple length scales, providing a generalizable framework for next-generation electrocatalysts.

## Teaser

A topology-engineered metallic-glass metamaterial catalyst integrates architecture and chemistry across scales, combining efficient fluid and gas transport, mechanical robustness and active interfaces for overall water splitting.

## INTRODUCTION

Hydrogen energy has emerged as a promising decarbonization vector, offering a carbon-neutral alternative to fossil fuels [1,2]. Among various hydrogen production routes, electrolytic water splitting stands out for its sustainability, yet its large-scale deployment remains limited by the intrinsic inefficiency of current electrocatalysts [3]. The enhancement of electrocatalytic efficiency relies on a tightly coupled multiscale design framework [4–8], spanning from atomic-level tuning of active sites [9], nano-level facilitation of charge transfer [10], micro-level control over reactant and product fluxes [11,12], to macro-level assurance of mechanical durability in operating devices [13]. However, simultaneously achieving multiscale design within a single catalyst system presents exceptional challenges due to the inherent conflicting structural features among functional requirements across different scales. For example, powder catalysts offer high specific surface areas and abundant active sites at the atomic/nano scale, yet they typically suffer from poor adhesion stability and inadequate charge-transfer pathways [14,15]. Conversely, structurally robust monolithic catalysts provide mechanical integrity at the macro scale but often sacrifice active site density and mass transport efficiency [16,17]. Therefore, developing a holistic design paradigm capable of reconciling these multiscale conflicts through coordinated architectural innovation has become an essential yet challenging pursuit in advanced catalyst engineering.

Nature provides numerous examples of architected materials that achieve multifunctional optimization through hierarchical organization [18,19]. Among them, marine echinoderms possess stereom structures consisting of highly porous and interconnected three-dimensional (3D) networks [20,21] that simultaneously provide mechanical support [22], efficient fluid transport [23] and attachment sites [23] for biological tissues. Previous studies have shown that the morphology of starfish ossicles (Fig. 1a i and ii) resembles diamond-type triply periodic minimal surface (TPMS) structures, while the medulla region of their spines [24] (Fig. 1a iii and iv) exhibits cellular features analogous to primitive- and diamond-type TPMS geometries [25,26]. Such naturally occurring systems illustrate how topology can be exploited to integrate structural robustness and transport efficiency within a single framework. Inspired by these characteristics, TPMS geometries were selected as the structural basis for catalyst scaffold design in this work. In such systems, strut geometry, channel connectivity and pore-size distribution govern key characteristics including mechanical robustness, fluid transport, electrolyte accessibility and gas-release behavior [27–29]. Owing to these topology-derived advantages, recent studies have proposed the concept of metamaterial catalysts, in which catalytic performance is enhanced through topology-controlled mass transport and mechanical optimization [28].

**Figure 1.**
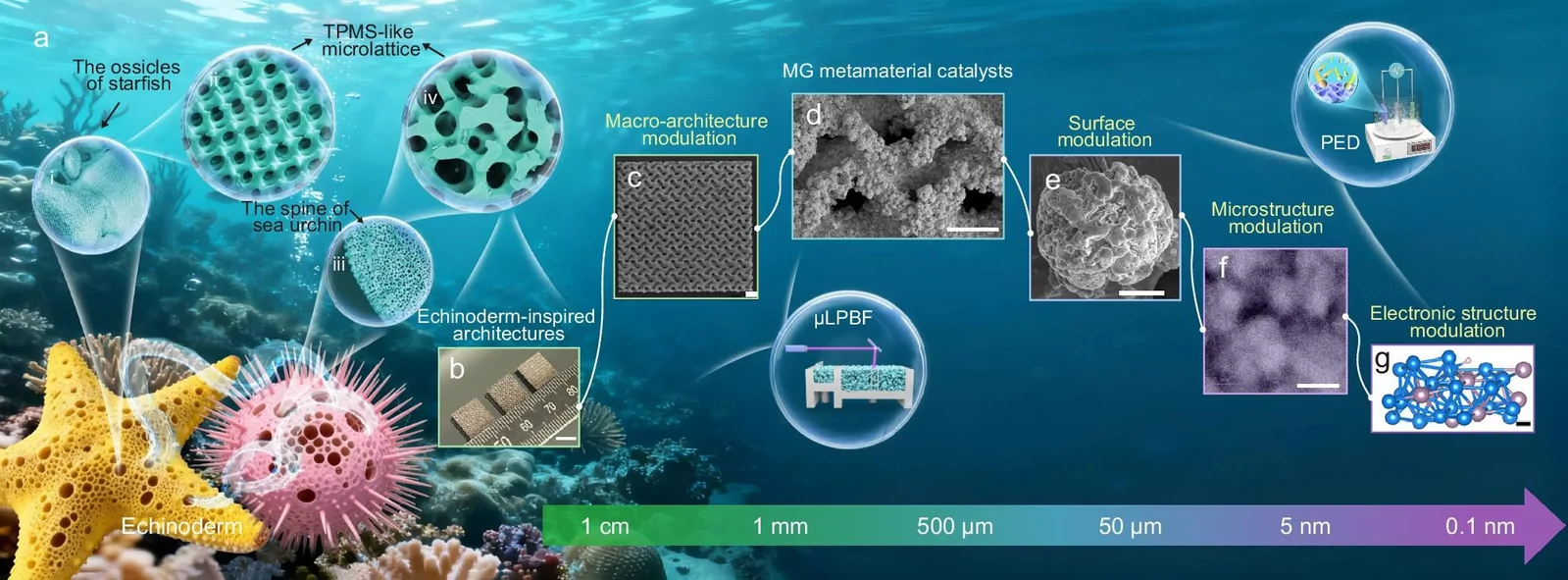
Multiscale design framework for topology-engineered metamaterial catalysts. (a) SEM images of sea urchin and starfish—(i) the microstructure of the ossicles of the starfish, (ii) localized magnified microstructure, (iii) the microstructure of the spine of the starfish and (iv) localized magnified microstructure. (b) Design concept of TPMS-based metallic glasses (MG) metamaterial scaffolds inspired by echinoderm stereom architectures. (c) SEM images of G-type MG microlattices fabricated by the additive manufacturing process. (d) SEM images of NiMo@Zr MMCs fabricated via the pulsed-electrodeposition (PED) process. (e) SEM images illustrating the hydrangea-like architecture of NiMo at the micro level. (f) HAADF-STEM images of the NiMo atomic structure. (g) Ni reaction sites in the atomic model of NiMo.

Despite these advances, most reported metamaterial catalysts remain primarily focused on architectural design at the macro/mesoscale [28,30]. The active catalytic phase is typically introduced through spray coating [28], dip coating [31] or catalyst-ink deposition [32], while the microstructural characteristics, interfacial structure and intrinsic catalytic activity of the deposited materials are rarely incorporated into the overall design strategy. As a result, the coupling between topology-controlled transport and micro-/nano-scale catalyst structure regulation remains largely unexplored. To fully exploit the potential of metamaterial catalysts, scaffold architecture and active phase must be considered simultaneously. Developing a multiscale metamaterial catalyst framework capable of integrating topology-engineered transport behavior with micro-/electronic-scale catalytic activity therefore represents an important yet largely unexplored direction.

Metallic glasses (MGs) provide a unique platform for constructing architected catalyst scaffolds owing to their exceptional strength, corrosion resistance and structural homogeneity [27]. Beyond their structural role, the intrinsic atomic disorder and nano-scale heterogeneity of amorphous materials have also been shown to influence catalytic behavior [33,34]. The nanoglass concept proposed by Gleiter and co-workers [35] has highlighted the unique role of abundant glass–glass interfaces in tailoring the structural and functional properties of amorphous materials. For example, Feng *et al.* reported that nano-scale interfacial heterogeneity in Ni–P MGs can effectively modulate local electronic structures and enhance electrocatalytic activity [36]. These findings suggest that amorphous heterointerfaces may provide an additional design dimension for extending metamaterial catalysts beyond topology-controlled transport optimization.

Guided by these insights, we propose a multiscale metamaterial catalyst design strategy that couples topology-controlled mass transport with nanoglass heterointerface-controlled catalytic activity within a single catalyst architecture. Specifically, multiscale metamaterial catalysts (denoted as NiMo@Zr MMCs) were constructed by integrating micro-laser powder bed fusion (μLPBF)-fabricated Zr-based MG TPMS scaffolds with pulsed-electrodeposition (PED)-derived NiMo nanoglass heterostructures. The TPMS microlattices fabricated via μLPBF (Fig. 1b), which closely resemble stereom geometries, are adopted as ideal catalyst frameworks. Following 3D printing, Ni and Mo species were uniformly deposited onto the surface of TPMS MG microlattices (Fig. 1c) through PED, forming the integrated NiMo@Zr MMC architecture illustrated in Fig. 1d. By tuning the PED parameters [37], we engineered a hydrangea-like morphology with complex grooves (Fig. 1e) to optimize wettability and bubble adhesion behavior. Meanwhile, a nano-scale disordered NiMo heterostructure was simultaneously formed (Fig. 1f), where compositional fluctuations at the Ni_78_Mo_22_/Ni_68_Mo_32_ interfaces modulate the electronic states via *d*-band center adjustment (Fig. 1g), thereby boosting the catalytic activity. Through the synergistic coupling of topology-controlled mass transport and interface-controlled catalytic activity, the resulting system establishes a unified structure–transport–activity framework spanning from the macro-scale architecture to the nano-scale catalytic interface. At each scale, the structure is precisely tailored to optimize the corresponding performance, and these individually controlled properties are integrated into a single catalyst through multiscale fabrication. Leveraging this multiscale design strategy, the resulting NiMo@Zr MMCs exhibit remarkable superhydrophilic and superaerophobic properties, as well as exceptional bifunctional catalytic performance for both hydrogen evolution reaction (HER) and oxygen evolution reaction (OER). Impressively, the system achieves an ultra-low cell voltage of 1.438 V at *j* = 100 mA cm^−2^ in alkaline media, along with outstanding durability exceeding 200 h for overall water splitting.

## RESULTS

### Mechanical and transport properties of topology-engineered MG scaffolds

Three types of TPMS structures with high surface area and favorable permeability—Gyroid (G), Fischer–Koch S (S) and Diamond (D)—were selected as architectural models for fabricating Zr-based MG scaffolds (Fig. S1). Zr-based MG lattices with varying geometries were successfully printed via μLPBF. X-ray diffraction (XRD) patterns (Fig. S2) revealed a broad halo diffraction peak in both the precursor powders and as-printed substrates, with no detectable crystalline Bragg reflections, confirming their fully amorphous structure. Each unit cell comprises solid shell phases and void channels with characteristic diameter *d* (Fig. 2a). Micro-computed tomography (micro-CT) reconstruction (Fig. 2b) verified the high geometric fidelity and printing quality of the structures. A single-channel scanning strategy was employed to control the shell thickness, which ranged from 90 to 150 µm—near the resolution limit of current LPBF systems. Porosity measurements derived from micro-CT (Table S1) revealed significant variations among the TPMS architectures: the S-Zr MG exhibited the highest relative density (RD) and the lowest porosity (∼52%), while the D and G MGs showed higher porosities (∼72%). Pore sizes of different types of substrates can be directly investigated based on the 3D reconstructed model (Fig. 2c). Although the D and G MGs exhibit similar overall porosity, the G MG clearly shows a larger average pore diameter and a broader pore size distribution, with a bimodal peak between 200 and 600 µm (Fig. S3), indicative of a hierarchical pore network. In contrast, the D structure shows a narrower pore size range of 100–500 µm, while the S structure features predominantly sub-300 µm pores, with a minimum diameter as small as 20 µm.

**Figure 2.**
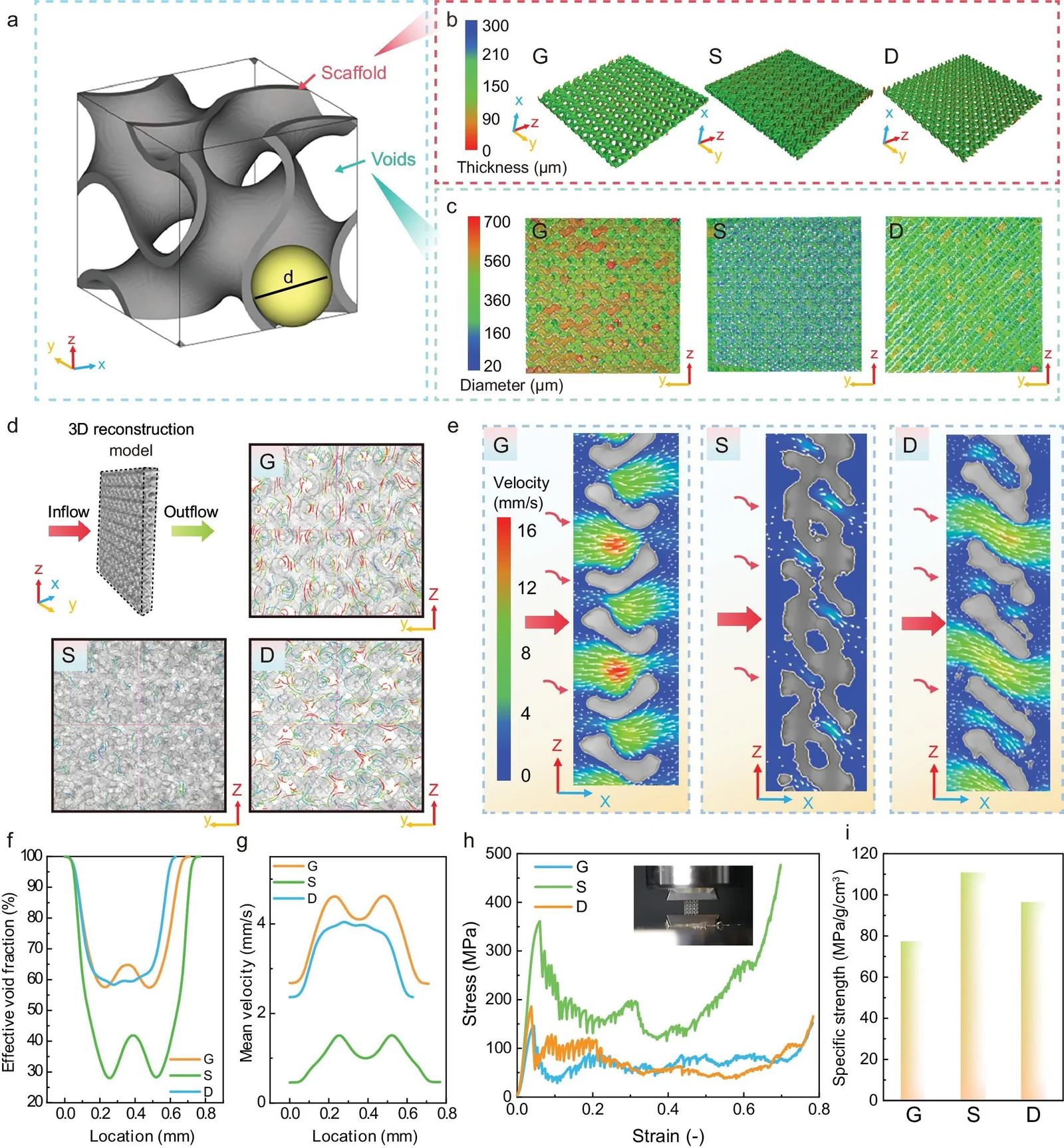
Morphological structure and mechanical performance of Zr-based MG TPMS lattice scaffolds. (a) Representative Gyroid unit cell’s characteristics. (b) Scaffold thickness derived from the micro-CT-reconstructed model. (c) Void diameter distribution derived from micro-CT-reconstructed. (d) Schematic illustration of the fluid flow simulation, showing flow direction (left to right) through the 3D-reconstructed lattice model. Velocity streamlines reveal distinct flow patterns across the three TPMS microlattices. (e) Two-dimensional velocity contour maps of a representative cross-section, showing localized flow acceleration zones and stagnation regions. (f) Axial variation of effective void fraction and (g) normalized average velocity along the primary flow direction (*x*-axis). (h) Stress–strain curves and (i) specific strength across the three TPMS microlattices.

Based on the 3D reconstruction model of the Zr-based MG scaffolds (Fig. 2d), the transport performance was analyzed to evaluate how different lattice architectures influence fluid flow behavior. Efficient mass transport minimizes concentration polarization and ensures stable electrochemical performance. The fluid flow behavior was simulated using a voxel-based finite volume method that directly incorporates the actual microstructural features obtained from CT scans, including pore shape and connectivity, thereby avoiding geometric simplification inherent to conventional mesh generation techniques. This method offers superior fidelity by inherently accounting for manufacturing-induced variations and imperfections in the additively fabricated substrates. Under standardized simulation conditions with a 1 Pa pressure difference between the inflow surface and outflow surface (Fig. S4), the G and D MG structures exhibited continuous and extended velocity streamlines (Fig. 2d and Fig. S5), indicative of higher permeability compared to the S MG structure, which exhibited shorter, more tortuous streamlines due to its lower porosity. Velocity contour maps of the cross-sections (Fig. 2e and Fig. S6) reveal localized increases in flow velocity within G MG, suggesting the presence of flow instabilities that promote enhanced mixing. In contrast, the D MG shows less velocity fluctuation across the structure (Fig. S7). Interestingly, despite D and G MGs having nearly identical overall porosities (∼28%), they display markedly different flow behaviors. G MG shows fluctuations in effective void fraction along the flow direction (Fig. 2f) with the highest mean flow velocity (Fig. 2g), whereas D MG maintains a relatively uniform distribution with a lower velocity. Although the overall porosity of S MG is ∼50%, the effective void fraction within the internal region drops to only ∼30% (Fig. 2f), resulting in a significantly lower mean velocity compared to the other two structures (Fig. 2g and Fig. S8). Furthermore, G MG demonstrated significantly enhanced vertical transport (*z*-direction velocity component; Fig. S9), enabling multidimensional reactant flow beyond the primary *x*–*y* plane, in contrast to the more uniform but less efficient flow patterns observed in D MG.

Damage-resistant, high-strength catalysts are essential for the long-term durability and operational stability of electrocatalytic systems. To assess the mechanical properties of the 3D-printed Zr-based MG scaffolds, uniaxial compression tests were conducted on 4 mm cubic samples. The resulting stress–strain curves (Fig. 2h) show high compressive strength and an extended stress plateau following the elastic region, indicating excellent damage tolerance of the printed structures. Among them, the S MG demonstrated the highest compressive strength, exceeding 300 MPa, which correlates with its maximum RD. When specific strength is considered (Fig. 2i), the S MG achieved the highest value of over 110 MPa kg^−1^ cm^−3^, surpassing many conventional metallic cellular materials. In contrast, the G MG exhibited a lower specific strength of 78 MPa kg^−1^ cm^−3^ due to its geometric characteristics. Notably, even the lowest performing G MG surpassed typical catalyst support requirements, such as nickel foam [38], by 10 times while retaining the porosity critical for mass transport.

Taken together, these results reveal that the superior performance of the G MG scaffold originates from its topology-enabled transport characteristics. Compared with the D and S MG architectures, the G structure possesses a continuously curved surface and a highly interconnected 3D channel network, which collectively promote higher permeability, faster electrolyte renewal and enhanced multidirectional mass transport. These characteristics are expected to facilitate reactant delivery and product removal throughout the scaffold while maximizing the accessibility of catalytic sites during electrolysis. More importantly, the advantages of the G architecture extend beyond geometric parameters such as porosity alone. Although the D and G MG structures exhibit comparable overall porosity, the G scaffold displays significantly higher average flow velocity, stronger velocity fluctuations and enhanced transport along the *z* direction. These features indicate more efficient electrolyte penetration and transport throughout the 3D framework. In addition, the continuous curvature of the G surface is expected to promote favorable wetting behavior and facilitate bubble detachment during gas-evolving reactions, thereby reducing local mass-transfer limitations. Consequently, the G architecture provides a more effective platform for catalyst utilization, leading to superior electrocatalytic performance compared with the D and S structures.

### Multiscale modulation of metamaterial catalysts

The tunable MG microlattices guarantee both structural toughness and efficient matter transport. To further elevate catalytic performance, we tailored the surface morphology at the micro scale and engineered the atomic structure at the nano scale. The surface morphology of catalysts serves as a crucial design parameter for regulating their wettability properties, where an optimal balance between superhydrophilicity (enhancing electrolyte wetting) and superaerophobicity (prompt bubble detachment) significantly improves the efficiency and durability of water-splitting systems [39,40]. The optimal catalyst scaffold G (Fig. 3b) and all other substrates (Fig. S10) were observed using scanning electron microscope (SEM), showing no internal defects such as pores or cracks apart from minor surface-adhered unmolten powder particles. These residual particles caused slight surface roughness, which beneficially increased the surface area and enhanced the adhesion of Ni and Mo atoms during the subsequent PED process. Based on the different scaffold architectures, the NiMo@MMCs are categorized into NiMo@G, NiMo@S and NiMo@D. A large number of uniform hydrangea-shaped NiMo particles, ranging in size from ∼60 to 100 µm (Fig. S11), were observed on NiMo@G (Fig. 3c) and across all substrate variants (NiMo@S and NiMo@D) (Fig. S12) after PED [29]. NiMo flowers grown on the scaffold surface create a hierarchically complex morphology that enables precise tuning of the surface wettability in the NiMo@Zr MMCs. The contact angle of the bare scaffold G is 103.65° after 300 s (Fig. 3d) with all the other substrates exhibiting a similar level of hydrophilicity (Fig. S13), whereas all NiMo@Zr MMCs exhibit superhydrophilicity (Fig. S14), with water droplets spreading completely upon contact, particularly for NiMo@G (Fig. 3e) that achieves instantaneous wetting (0° contact angle within just 35 ms). Concurrently, these NiMo@Zr MMCs demonstrate remarkable superaerophobic characteristics in aqueous environments. Specifically, the bubble contact angle (CA) of scaffold G is 155° (Fig. 3f) whereas the NiMo@G shows the ultra-hydrophobicity (CA > 169°) (Fig. 3g). The bubble contact angles significantly improved to varying extents after surface modification of all the different substrates via PED (Figs S15 and S16).

**Figure 3.**
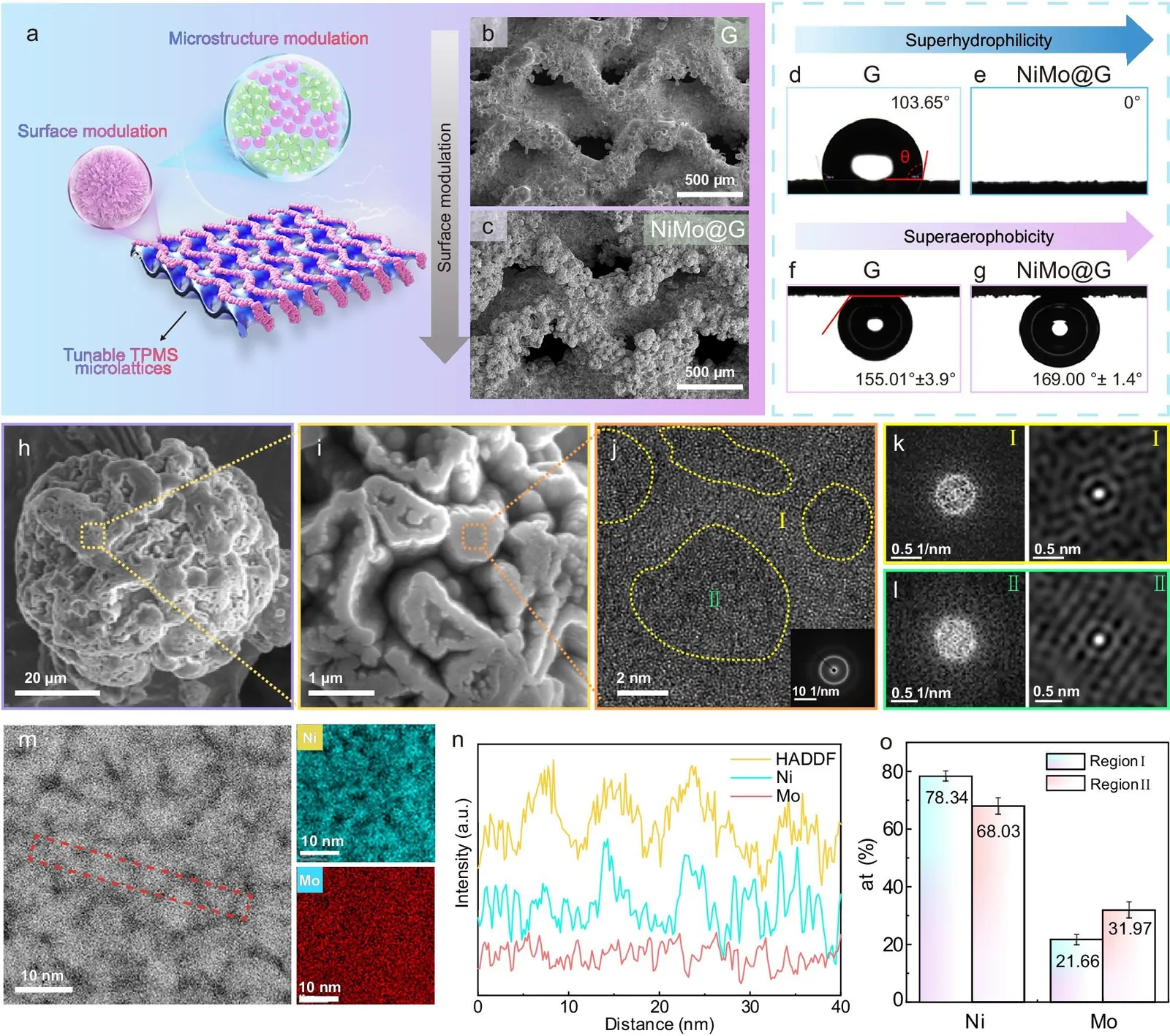
Wettability and bubble adhesion characteristics and micro–nano structure of NiMo@Zr MMCs. (a) Multiscale structure modulation in the integrated metamaterial catalysts. (b) SEM images comparing the surface morphology of as-printed G MG scaffold with (c) corresponding NiMo@G after PED. (d) Images of water contact angles of 103.65° when water droplets are dropped on the surfaces of the G MG. (e) The wetting behavior demonstrating superhydrophilic properties of NiMo@G. (f) Under electrolyte conditions, quantitative bubble adhesion measurements show that the G MG without PED treatment exhibits a contact angle of 155.0° ± 3.9°, while (g) NiMo@G demonstrates a significantly enhanced superaerophobic surface with a contact angle of 169.0° ± 1.4°. (h and i) SEM images illustrating the hydrangea-like architecture of NiMo grown on the MG scaffold via PED. (j) HRTEM image with an insert SAED pattern, revealing two distinct regions: (I) interfacial area and (II) clustered structure. (k and l) FFT patterns and autocorrelation images of region I and region II from panel (j), respectively, confirming the coexistence of structural inhomogeneity. (m) Corresponding EDX elemental maps for Ni and Mo and the associated HAADF-STEM image revealing nano-scale compositional heterogeneity. (n) Line scan of elemental distribution across a selected region from panel (i) confirming the spatial variation of Ni and Mo. (o) Atomic ratios of Ni and Mo from two distinct regions identified in the panel (j).

A closer inspection (Fig. 3h–i) reveals the detailed morphology of the NiMo particles, characterized by a highly wrinkled and uneven surface, which increases the specific surface area and exposes more active catalytic sites. The bare scaffolds, without NiMo deposition, exhibited minimal catalytic activity (Fig. S17). To further boost catalytic activity, we modulate the microstructure of the NiMo particles. While elemental mapping demonstrates macro-scale uniformity in Ni and Mo distribution across the entire flower-like structures (Fig. S18), nano-scale analysis through high-angle annular dark-field scanning transmission electron microscopy (HAADF-STEM) reveals distinct contrast variations (Fig. S19a), indicating phase separation. The overall selected area electron diffraction (SAED) pattern (Fig. 3j insert) exhibits a halo ring, indicative of an amorphous structure. High-resolution transmission electron microscope (HRTEM) analysis reveals a disordered region (region I) and a more loosely arranged, medium-range ordered atomic cluster (region II) (Fig. 3j and Fig. S19b). The interface region (I) exhibits a highly disordered maze-like structure with inner circular fringes (Fig. 3k) and a featureless fast Fourier transform (FFT) pattern, whereas the cluster region (II) displays clear crystalline fringes (Fig. 3l) with distinct diffraction spots in the FFT pattern. HAADF-STEM reveals distinct contrast variations (Fig. 3m), indicating phase separation into Ni-rich (bright) and Mo-rich (dark) amorphous domains. Energy dispersive X-ray spectroscopy (EDX) mapping confirms this structural heterogeneity, showing significant local Ni concentration variations while Mo maintains a relatively uniform distribution. Integrated compositional line scans (Fig. 3n) quantitatively verify that the brighter STEM regions correspond to Ni_78_Mo_22_ domains, while darker areas exhibit Ni_68_Mo_32_ stoichiometry—a 10% compositional difference statistically confirmed by EDX mapping (Fig. 3o and Fig. S20). Atomic-scale characterization reveals that the PED-fabricated NiMo hydrangea structures form an amorphous chemical heterostructure, featuring numerous interfaces between compositionally distinct domains (Ni_78_Mo_22_ and Ni_68_Mo_32_). These interfaces are believed to offer abundant highly active sites for catalytic reactions.

### Excellent bi-functional performance with HER and OER and mechanism

We further evaluated the electrocatalytic performance of the NiMo@Zr MMCs for water electrolysis in 1 M KOH, as shown in Fig. 4. NiMo@G (Fig. 4a) exhibits a remarkably low HER overpotential of 26 mV at 10 mA cm^−2^, significantly outperforming NiMo@S (59 mV), NiMo@D (81 mV) and commercial Pt/C (55 mV). Tafel analysis (Fig. 4c) reveals NiMo@G’s exceptional HER kinetics with a record-low 27.91 mV dec^−1^ slope, outperforming NiMo@S (76.79 mV dec^−1^), NiMo@D (91.94 mV dec^−1^) and commercial Pt/C (33.42 mV dec^−1^) by 2.7–3.3 times, demonstrating its superior charge-transfer efficiency. NiMo@G exhibits outstanding HER performance at industrially relevant current densities (Fig. 4e), requiring only 79 and 126 mV of overpotential to achieve 100 and 300 mA cm^−2^, respectively. This superior activity under high-current conditions highlights its strong potential for practical water-splitting applications. We further assessed the OER performance of the NiMo@G, as shown in Fig. 4b. All samples exhibit a distinct oxidation peak during the OER process, corresponding to the oxidation of Ni species that facilitate oxygen evolution. This observation confirms the excellent intrinsic electrocatalytic activity of the NiMo@Zr MMCs. To accurately evaluate the overpotential and minimize the influence of the oxidation peak, cyclic voltammetry (CV) was employed. NiMo@G demonstrates exceptional OER performance with an ultra-low overpotential of 155 mV at 10 mA cm^−2^, significantly outperforming NiMo@S (181 mV), NiMo@D (204 mV) and commercial IrO_2_ (Fig. 4b). Tafel analysis (Fig. 4d) reveals NiMo@G’s superior OER kinetics with a record-low Tafel slope of 31.08 mV dec^−1^, outperforming NiMo@S (42.79 mV dec^−1^), NiMo@D (54.90 mV dec^−1^) and IrO_2_ (128.99 mV dec^−1^), demonstrating significantly faster reaction rates. Figure 4e presents the overpotentials at various current densities (10, 50, 100 and 300 mA cm^−2^). Notably, NiMo@G consistently delivers the lowest overpotentials across all current densities, especially in industrial-grade currents. For example, NiMo@G demonstrates superior performance with significantly lower overpotentials (204 mV @ 100 mA cm^−2^; 261 mV @ 300 mA cm^−2^), representing a 18%–23% reduction in overpotential across industrial-relevant current densities compared with other samples. Compared with crystallized G scaffolds and Ni foam controls, NiMo@G exhibits superior catalytic performance (Fig. S21). This indicates that the enhanced activity originates not only from the active NiMo phase or surface-area enlargement, but also from the synergistic contributions of the amorphous MG scaffold, topology-engineered mass transport enabled by the TPMS architecture and intrinsically active NiMo nanoglass interfaces. The electrochemically active surface area (ECSA) was evaluated by extracting the double-layer capacitance (*C*_dl_) from the CV curves in the non-Faradaic region (Fig. 4f). NiMo@S, with a *C*_dl_ of 36.45 mF cm^−2^, exposes more active sites than NiMo@D (*C*_dl_ = 4.65 mF cm^−2^), leading to higher catalytic activity. Remarkably, NiMo@G exhibits the highest active site density, with a *C*_dl_ of 105.31 mF cm^−2^, which correlates with its outstanding catalytic performance. Furthermore, ECSA-normalized HER and OER polarization curves confirm that NiMo@G remains highly competitive after normalization (Figs S22 and S23). Electrochemical impedance spectroscopy results for NiMo@G (Fig. S24) show a smaller Nyquist plot radius compared to both NiMo@S and NiMo@D, indicating lower charge-transfer resistance and superior electron transport, thereby enhancing catalytic performance.

**Figure 4.**
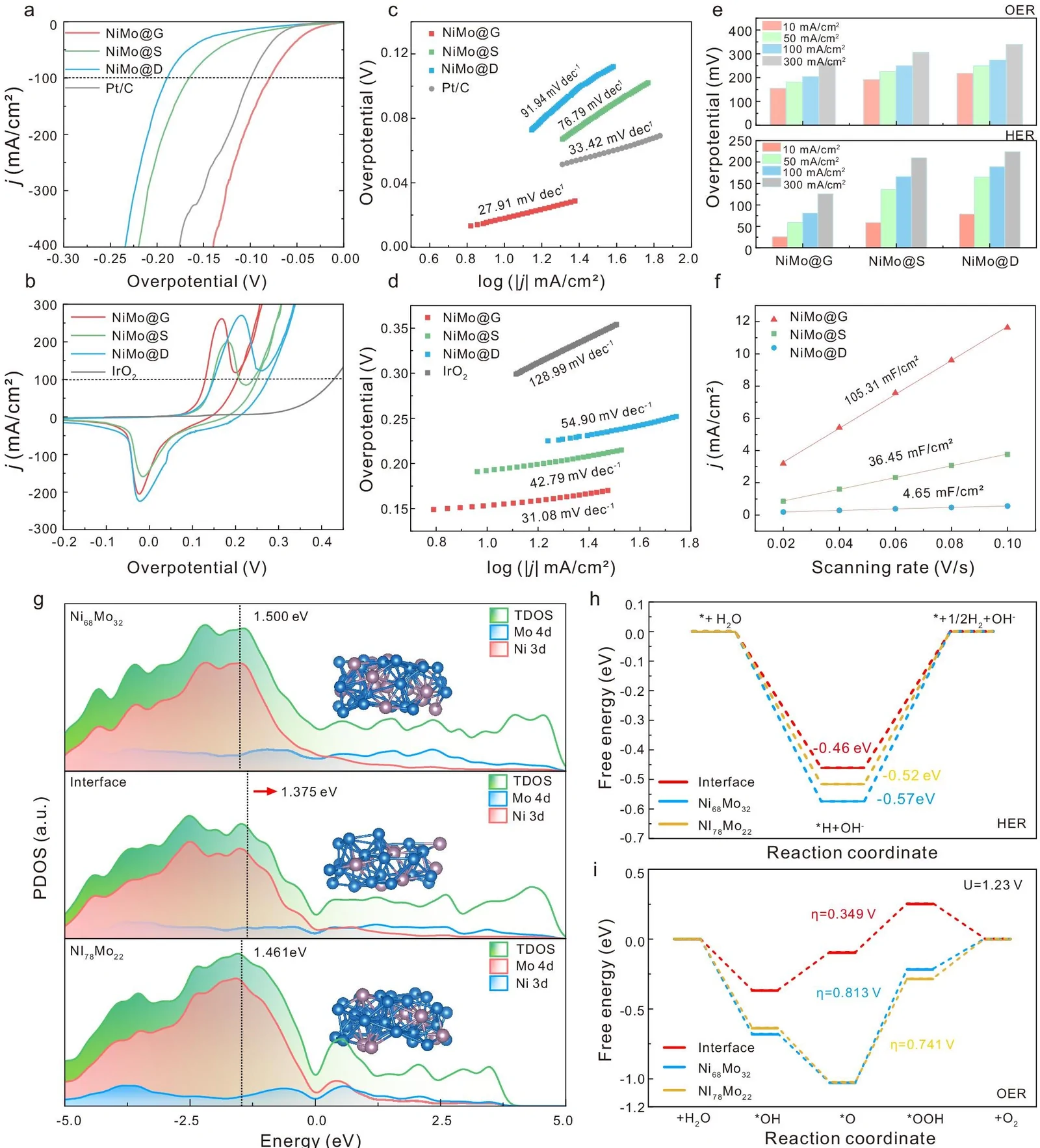
HER and OER performance and DFT simulations of the NiMo@Zr MMCs. (a) HER polarization curves and (b) OER polarization curves of different NiMo@Zr MMCs. (c) Tafel plots of NiMo@Zr MMCs for HER. (d) Tafel plots of NiMo@Zr MMCs for OER. (e) Overpotentials at 10, 50, 100 and 300 mA cm^−2^ for OER and HER. (f) The *C*_dl_ values of NiMo@Zr MMCs. (g) PDOS for the Ni and Mo active sites in different NiMo MGs (Ni_78_Mo_22_, interface and Ni_68_Mo_32_). The black solid lines highlight the corresponding total *d*-band center. (h) Comparison of the Δ*G* profiles for Ni catalytic sites in NiMo MGs for HER by DFT calculations. (i) Comparison of the Δ*G* profiles for Ni catalytic sites in NiMo MGs for OER by DFT calculations.

To better understand the origin of the exceptional OER and HER performance of the chemically heterogeneous NiMo MG catalysts, we employed density functional theory (DFT) calculations to evaluate their free energy profiles and electronic structures. Large-scale amorphous Ni_78_Mo_22_ and Ni_68_Mo_32_ models were first generated through melt–quench molecular dynamics simulations (Fig. S25), followed by the construction of an amorphous Ni_78_Mo_22_/Ni_68_Mo_32_ interfacial structure (Fig. 4g and ‘Methods’ section). To explore the electronic structures of the three distinct NiMo MGs, the partial density of states (PDOS) for Ni and Mo, as well as the total density of states, were calculated, as shown in Fig. 4g. Catalytic activity is predominantly controlled by the *d*-band center’s position relative to the Fermi level (*E*_F_), where closer proximity enhances adsorbate binding via strengthened *d*-orbital interactions. The Ni 3d *d*-band center shifts markedly closer to the *E*_F_ (1.375 eV) at the interface compared to both Ni_68_Mo_32_ (1.500 eV) and Ni_78_Mo_22_ (1.461 eV) MGs, indicating enhanced catalytic activity through strengthened adsorbate interactions at interfacial Ni sites. However, the *d*-band center of Mo remains relatively unchanged, suggesting that the variation in catalytic activity is primarily associated with the Ni 3d orbitals.

To account for the intrinsic structural heterogeneity of amorphous materials, 10 representative local environments (Fig. S26) were randomly sampled from each region (Ni_78_Mo_22_, Ni_68_Mo_32_ and the interfacial region) for statistical HER calculations. As shown in Fig. 4h and Fig. S27, the interfacial region exhibits consistently more favorable hydrogen adsorption behavior than the two amorphous phases. The statistical distribution of ΔG_H* further indicates that the enhanced HER activity is a general characteristic of the amorphous–amorphous interface rather than a single optimized adsorption site (Figs S26 and S28). In OER, the interface structure reduces the rate-determining step energy barrier (G_*O_–G_*OOH_), yielding a substantially lower overpotential (*η* = 0.349 V) compared to Ni_68_Mo_32_ (*η* = 0.813 V) and Ni_78_Mo_22_ (*η* = 0.741 V), as shown in Fig. 4i and Fig. S29. These results suggest that the abundant amorphous–amorphous interfaces within the NiMo nanoglass heterostructure provide electronically favorable catalytic environments, thereby contributing to the enhanced bifunctional electrocatalytic performance.

### Evaluation of the overall water-splitting performance

The NiMo@G demonstrate exceptional HER and OER performance, rendering them highly suitable for overall water-splitting applications. A dual-electrode electrolyzer was assembled using NiMo@G as both the anode and cathode. The polarization curves (Fig. 5a) reveal that this system achieves current densities of 100 and 500 mA cm^−2^ at ultra-low cell voltages of 1.438 and 1.707 V, respectively—significantly lower than that of the benchmark IrO_2_//Pt/C system (1.783 V @ 100 mA cm^−2^). High-speed imaging of bubble dynamics at 100 mA cm^−2^ clearly demonstrates the operational advantages of this design: while the bubble-release behaviors of conventional porous IrO_2_ electrodes suffer from persistent bubble adhesion and coalescence into large (>500 μm) gas pockets that block active sites (Fig. 5b and Video S1), the NiMo@G catalyst enables rapid release of small (∼70 μm) bubbles (Fig. 5c and Video S2). Stability testing via chronopotentiometry (Fig. 5d) confirms the excellent durability of NiMo@G, with negligible current density loss over 200 h of continuous operation at 1.438 V. Interestingly, the overall water-splitting performance slightly improved after the 200-h test, as shown by the enhanced polarization curve (pink line in Fig. 5a). Post-OER X-ray photoelectron spectroscopy (XPS) analyses reveal the disappearance of the peak of Ni^0^ together with the emergence of Ni^3+^ peak. Meanwhile, the O 1s spectra show the appearance of OH^−^ peak, suggesting the formation of NiOOH-rich surface phases during prolonged electrolysis (Fig. S30). The formation of NiOOH has been widely recognized as the catalytically active phase for alkaline OER and is therefore likely responsible for the enhanced catalytic activity after long-term operation [41–43]. In addition, the Mo 3d spectra shift toward higher binding energies after the OER test, indicating the formation of higher-valent Mo species, which may act synergistically with the Ni^3+^ to further promote the OER kinetics. Meanwhile, post-stability SEM and EDX characterizations confirm that both the hierarchical NiMo coating and the underlying MG scaffold remain structurally intact without obvious morphology degradation or elemental segregation after 200 h electrolysis (Fig. S31), demonstrating the excellent durability of the multiscale catalyst architecture.

**Figure 5.**
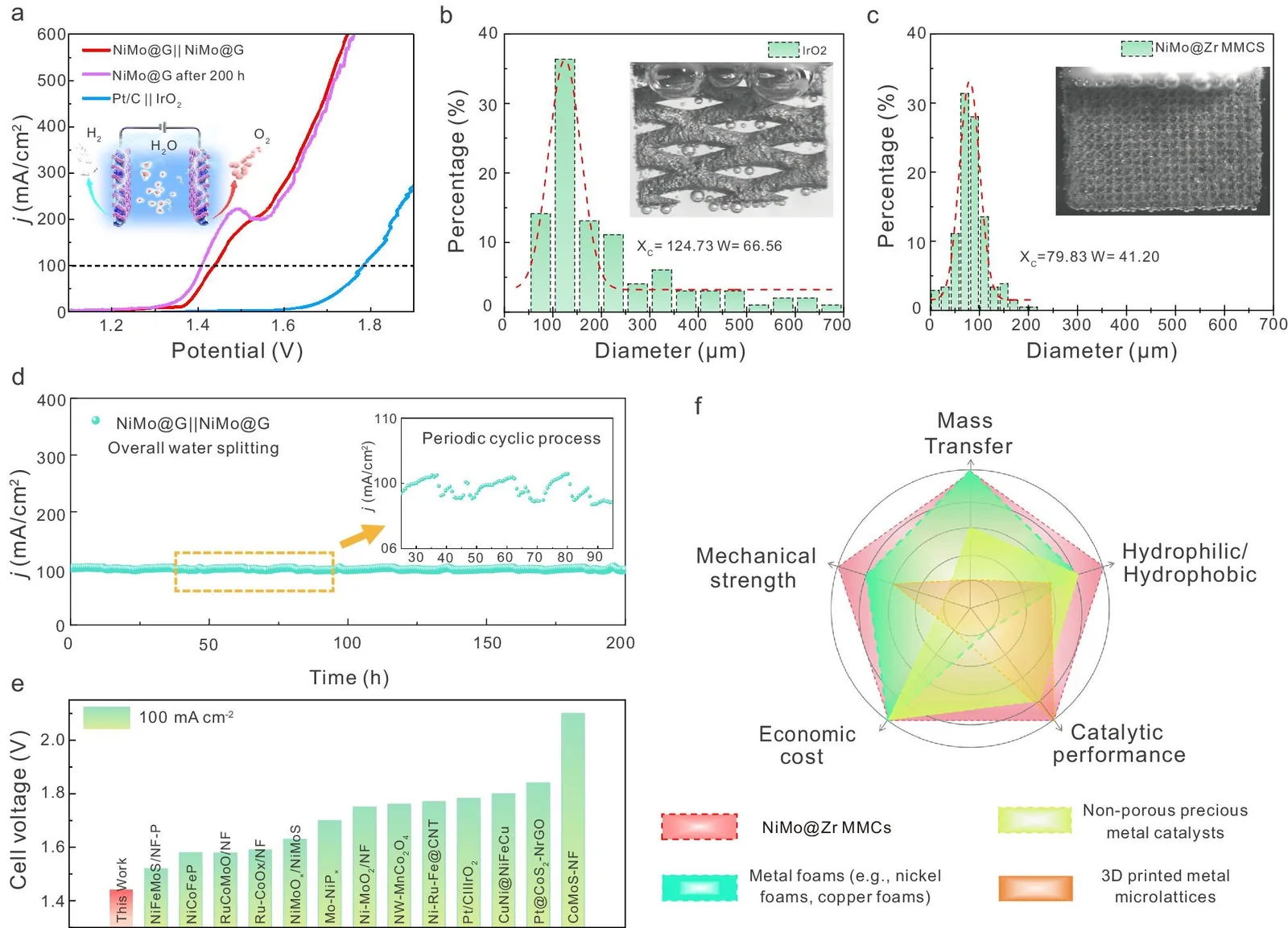
Overall water-splitting performance of NiMo@G. (a) Overall water-splitting-polarization curves before and after a 200-h stability test, demonstrating performance retention. (b) Bubble size distribution of the IrO_2_ electrode and (c) NiMo@G electrode. (d) Chronopotentiostatic stability test at 100 mA cm^−2^, showing minimal voltage fluctuation over 200 h of continuous operation. (e) Comparison of cell voltages at 100 mA cm^−2^, benchmarking NiMo@G against state-of-the-art catalysts reported in literature (data in Table S2). (f) The radar chart comparing the properties of the different types of electrolytes (more details presented in Table S3).

Furthermore, during the initial operation, the NiMo@G//NiMo@G configuration demonstrated one of the lowest cell voltages among reported catalysts at high current densities (1.438 V @ 100 mA cm^−2^; Fig. 5e). This work highlights the synergistic combination of 3D printing technology, which enables precise geometric control, flexible design capabilities and tunable mechanical/mass-transfer properties, with PED that addresses the limitations in microstructural control. Compared to conventional porous metal catalysts, precious metal catalysts and metal microlattices, the NiMo@G MG metamaterial catalysts uniquely combine the multiple advantages of structural strength, super hydrophobic and superhydrophilicity, low economic costs and extraordinary catalytic activity (Fig. 5f), establishing a new paradigm for designing high-performance electrocatalytic system. The proposed multiscale metamaterial catalyst framework is not restricted to the NiMo system investigated in this work. Preliminary experiments (Fig. S32) on NiMoFeCo coatings deposited on the same TPMS MG scaffold reveal distinct amorphous/nanocrystalline heterostructures together with promising electrocatalytic performance. These observations suggest that the framework can be readily extended to a broader range of catalyst compositions.

## CONCLUSION

In summary, this work proposes a multiscale metamaterial catalyst design strategy that integrates topology-engineered transport pathways, architected MG scaffolds and nanoglass heterointerface-regulated catalytic activity within a single catalyst architecture. Drawing inspiration from the stereom architecture of echinoderms, we translated their unique coupling of mechanical resilience and matter-transport efficiency into MG scaffolds with TPMS geometries. To achieve hierarchical control over structure and composition, PED was employed to integrate NiMo amorphous particles onto TPMS MG scaffolds. This strategy enables concurrent optimization of macro-scale mechanical robustness, micro-scale surface wettability and atomic-scale catalytic activity. Among the architectures, the Gyroid-based NiMo@Zr MMCs exhibited superior compressive strength (>300 MPa), enhanced mass transfer and outstanding bifunctional catalytic performance, requiring only 1.438 V to reach 100 mA cm^−2^ with stable operation for 200 h. More importantly, this work extends the concept of metamaterial catalysts beyond conventional topology-controlled transport optimization toward the integration of micro-/nano-scale catalyst structure regulation. The proposed multiscale design framework provides a generalizable pathway for designing next-generation metamaterial catalysts.

## Supplementary Material

nwag442_Supplemental_Files

## References

[1] Chu S, Majumdar A. Opportunities and challenges for a sustainable energy future. Nature 2012; 488: 294–303. 10.1038/nature11475 22895334

[2] Huang Y, Zhu M, Huang Y et al. Multifunctional energy storage and conversion devices. Adv Mater 2016; 28: 8344–64. 10.1002/adma.201601928 27434499

[3] McKone JR, Marinescu SC, Brunschwig BS et al. Earth-abundant hydrogen evolution electrocatalysts. Chem Sci 2014; 5: 865–78. 10.1039/C3SC51711J

[4] Tang B, Yang X, Kang Z et al. Crystallized RuTe_2_ as unexpected bifunctional catalyst for overall water splitting. Appl Catal B Environ 2020; 278: 119281. 10.1016/j.apcatb.2020.119281

[5] Amiripour F, Ghasemi S. Construction of flower-like nanoassembly P-doped MOF-derived MoS_2_/Co_9_S_8_ grown on hexagonal microporous alumino-silicate framework for overall water splitting in alkaline media. Fuel 2023; 346: 128299. 10.1016/j.fuel.2023.128299

[6] Wang C, Shao X, Pan J et al. Redox bifunctional activities with optical gain of Ni_3_S_2_ nanosheets edged with MoS_2_ for overall water splitting. Appl Catal B Environ 2020; 268: 118435. 10.1016/j.apcatb.2019.118435

[7] Yao Y, Dong Q, Brozena A et al. High-entropy nanoparticles: synthesis–structure–property relationships and data-driven discovery. Science 2022; 376: eabn3103. 10.1126/science.abn3103 35389801

[8] Yan Y, Chen Y, Shao M et al. Controlled synthesis of self-supportive hydrangea-shaped Ni-based hydroxide/phosphide for efficient overall water splitting and full-pH hydrogen evolution. Fuel 2024; 367: 131551. 10.1016/j.fuel.2024.131551

[9] Li S, Xin Z, Luo Y et al. Recent advances in the development of single-atom catalysts for oxygen evolution reaction. Int J Hydrogen Energy 2024; 82: 1081–100. 10.1016/j.ijhydene.2024.08.026

[10] Kazemi A, Manteghi F, Tehrani Z. Metal electrocatalysts for hydrogen production in water splitting. ACS Omega 2024; 9: 7310–35. 10.1021/acsomega.3c07911 PMC10882616 38405471

[11] Qadeer MA, Zhang X, Farid MA et al. A review on fundamentals for designing hydrogen evolution electrocatalyst. J Power Sources 2024; 613: 234856. 10.1016/j.jpowsour.2024.234856

[12] Han C, Yuan Y, Chen G et al. Interface engineering induced multi-scale self-assembly NiFe-LDH heterostructures for high-performance water electrolysis. ChemSusChem 2024; 17: e202400812. 10.1002/cssc.202400812 38847216

[13] Wu D, Zhou J, Li Y. Mechanical strength of solid catalysts: recent developments and future prospects. AIChE J 2007; 53: 2618–29. 10.1002/aic.11291

[14] Andronescu C, Barwe S, Ventosa E et al. Powder catalyst fixation for post-electrolysis structural characterization of NiFe layered double hydroxide based oxygen evolution reaction electrocatalysts. Angew Chem Int Ed 2017; 56: 11258–62. 10.1002/anie.201705385 28649797

[15] Valinton JAA, Lin M-Y, Tsai C-H et al. A robust inorganic binder against corrosion and peel-off stress in electrocatalysis. Chem Sci 2024; 15: 16966–76. 10.1039/D4SC04088K PMC11423651 39345769

[16] Govender S, Friedrich HB. Monoliths: a review of the basics, preparation methods and their relevance to oxidation. Catalysts 2017; 7: 62. 10.3390/catal7020062

[17] Wang Q, Sui J, Li L et al. Recent advances in regulating ceramic monolithic catalyst structure for preferential oxidation of CO in H_2_. Molecules 2024; 29: 3481. 10.3390/molecules29153481 PMC11314092 39124886

[18] Gladman AS, Matsumoto EA, Nuzzo RG et al. Biomimetic 4D printing. Nat Mater 2016; 15: 413–8. 10.1038/nmat4544 26808461

[19] Feng C, Zhang W, Deng C et al. 3D printing of lotus root-like biomimetic materials for cell delivery and tissue regeneration. Adv Sci 2017; 4: 1700401. 10.1002/advs.201700401 PMC5737106 29270348

[20] Gorzelak P, Zamora S. Stereom microstructures of cambrian echinoderms revealed by cathodoluminescence (CL). Palaeontol Electron 2013; 16: 1–17.

[21] Perricone V, Cesarano P, Deb M et al. The microarchitectural variability in the echinoid skeleton: a 3D geometrical and stiffness characterization of *Paracentrotus lividus*. R Soc Open Sci 2025; 12: 241439. 10.1098/rsos.241439 PMC12187406 40568548

[22] Strathmann RR . The role of spines in preventing structural damage to echinoid tests. Paleobiology 1981; 7: 400–6. 10.1017/S0094837300004693

[23] Gorzelak P . Functional Micromorphology of the Echinoderm Skeleton. Cambridge: Cambridge University Press, 2021, 5–10. 10.1017/9781108893886

[24] Klang K, Nickel KG. The plant-like structure of lance sea urchin spines as biomimetic concept generator for freeze-casted structural graded ceramics. Biomimetics 2021; 6: 36. 10.3390/biomimetics6020036 PMC8261639 34072652

[25] Yang T, Jia Z, Wu Z et al. High strength and damage-tolerance in echinoderm stereom as a natural bicontinuous ceramic cellular solid. Nat Commun 2022; 13: 6083. 10.1038/s41467-022-33712-z PMC9568512 36241635

[26] Yu H, Li J, Hou Z et al. Investigation on the effect of the multilayered porous structure of sea urchin skeleton on its mechanical behavior. J Bionic Eng 2020; 17: 469–83. 10.1007/s42235-020-0038-z

[27] Yang C, Ding J, Qu S et al. High strength bioinspired cellular metallic glasses with excellent energy absorption. Acta Mater 2025; 285: 120688. 10.1016/j.actamat.2024.120688

[28] Zhang L, Liu H, Song B et al. Wood-inspired metamaterial catalyst for robust and high-throughput water purification. Nat Commun 2024; 15: 2046. 10.1038/s41467-024-46337-1 PMC10917756 38448407

[29] Feng J, Fu J, Yao X et al. Triply periodic minimal surface (TPMS) porous structures: from multi-scale design, precise additive manufacturing to multidisciplinary applications. Int J Extrem Manuf 2022; 4: 022001. 10.1088/2631-7990/ac5be6

[30] Zhu C, Qi Z, Beck VA et al. Toward digitally controlled catalyst architectures: hierarchical nanoporous gold via 3D printing. Sci Adv 2018; 4: eaas9459. 10.1126/sciadv.aas9459 PMC6118649 30182056

[31] Bogdan E, Michorczyk P. 3D printing in heterogeneous catalysis: the state of the art. Materials 2020; 13: 4534. 10.3390/ma13204534 PMC7601972 33066083

[32] Chen L, Zhou S, Li M et al. Catalytic materials by 3D printing: a mini review. Catalysts 2022; 12: 1081. 10.3390/catal12101081

[33] Jian X, Zhang W, Yang Y et al. Amorphous Cu–W alloys as stable and efficient electrocatalysts for hydrogen evolution. ACS Catal 2024; 14: 2816–27. 10.1021/acscatal.3c05820

[34] Wu K, Chu F, Meng Y et al. Cathodic corrosion activated Fe-based nanoglass as a highly active and stable oxygen evolution catalyst for water splitting. J Mater Chem A 2021; 9: 12152–60. 10.1039/D1TA00769F

[35] Gleiter H . Nanoglasses: a new kind of noncrystalline materials. Beilstein J Nanotechnol 2013; 4: 517–33. 10.3762/bjnano.4.61 PMC3778333 24062978

[36] Pei C, Chen S, Zhao T et al. Nanostructured metallic glass in a highly upgraded energy state contributing to efficient catalytic performance. Adv Mater 2022; 34: 2200850. 10.1002/adma.202200850 35429007

[37] Lee SA, Yang JW, Choi S et al. Nanoscale electrodeposition: dimension control and 3D conformality. Exploration 2021; 1: 20210012. 10.1002/EXP.20210012 PMC10191033 37323687

[38] Rajak DK, Gupta M. Applications of metallic foams. In:* An Insight Into Metal Based Foams: Processing, Properties and Applications*. Singapore: Springer Singapore, 2020, 21–37. 10.1007/978-981-15-9069-6 10.1007/978-981-15-9069-6_2

[39] Shan X, Liu J, Mu H et al. An engineered superhydrophilic/superaerophobic electrocatalyst composed of the supported CoMoS chalcogel for overall water splitting. Angew Chem Int Ed 2020; 59: 1659–65. 10.1002/anie.201911617 31639242

[40] Liao Y, Deng S, Qing Y et al. Hierarchically wood-derived integrated electrode with tunable superhydrophilic/superaerophobic surface for efficient urea electrolysis. J Energy Chem 2023; 76: 566–75. 10.1016/j.jechem.2022.10.007

[41] Zhu J, Qian J, Peng X et al. Etching-induced surface reconstruction of NiMoO_4_ for oxygen evolution reaction. Nano-Micro Lett 2023; 15: 30. 10.1007/s40820-022-01011-3 PMC9829944 36624193

[42] Gallenberger J, Moreno Fernández H, Alkemper A et al. Stability and decomposition pathways of the NiOOH OER active phase of NiO_x_ electrocatalysts at open circuit potential traced by ex situ and in situ spectroscopies. Catal Sci Technol 2023; 13: 4693–700. 10.1039/D3CY00674C

[43] Klaus S, Cai Y, Louie MW et al. Effects of Fe electrolyte impurities on Ni(OH)_2_/NiOOH structure and oxygen evolution activity. J Phys Chem C 2015; 119: 7243–54. 10.1021/acs.jpcc.5b00105

