# Supplementary material for: Topology-engineered metallic glass metamaterial-supported NiMo nanoglass catalysts for ultra-efficient water splitting
Source: Natl Sci Rev. 2026 Jul 17;13(17):nwag442. doi: 10.1093/nsr/nwag442 (PMC13639190; doi:10.1093/nsr/nwag442)
Supplement: Supplementary file 1 [file nwag442_supplemental_files.zip › Supplementary data.pdf]

Supplemental Information for

Topology-engineered metallic glass metamaterial-supported NiMo nanoglass catalysts for ultra-efficient water splitting

Congrui Yang[1,3,†], Chaoqun Pei[1,4,†], Haibo Ke[1,2,*] Shilong Sun[1], Huaping Zhang[1,2], Di Ouyang[5], Lei Zhang[6], Huaping Ding[1], Yongyun Zhang[1], Yuyang Qian[1], Baoan Sun[1], K.C. Chan[3,*] and Wei-Hua Wang[1,2,*]

[1]Songshan Lake Materials Laboratory, Dongguan 523808, China;

[2]Dongguan Institute of Materials Science and Technology, Chinese Academy of Sciences, Dongguan 523808, China;

[3]Research Institute for Advanced Manufacturing, Department of Industrial and Systems Engineering, The Hong Kong Polytechnic University, Hong Kong, China;

[4]Shenzhen Key Laboratory of High Performance Nontraditional Manufacturing, College of Mechatronics and Control Engineering, Shenzhen University, Shenzhen 518060, China;

[5]Department of Metallic Materials Engineering, College of Materials Science and Engineering, Wuhan University of Technology, Wuhan 430074, China;

[6]Department of Mechanical Engineering, City University of Hong Kong, Hong Kong, China

***Corresponding authors.** E-mails: kehaibo@sslab.org.cn; kc.chan@polyu.edu.hk; whw@iphy.ac.cn

[†]Equally contributed to this work.

Table of Contents

Methods
Supplementary Figures S1-S31
Supplementary Tables 1-3
Supplementary Videos 1-2
Supplementary References

METHODS AND METHODS

Geometric modeling and fabrication of Zr-based MG scaffolds

Gas-atomized $Zr_{60.14}Cu_{22.31}Fe_{4.85}Al_{9.7}Ag_3$ metallic glass (MG) powders were employed as feedstock for substrate fabrication via the micro-laser powder bed fusion (μLPBF) process. The powders, with particle sizes ranging from 0 to 53 μm, exhibited excellent sphericity, uniform size distribution, and high surface quality, making them well-suited for LPBF processing. MG substrates were fabricated using a BLT-S200GX μLPBF system, which differs from standard LPBF equipment by offering a refined laser spot size of just 30 μm, enabling the production of high-resolution components. The reduced laser spot size and melt-pool dimensions allow the fabrication of architected MG scaffolds with characteristic feature sizes below 100 μm and excellent geometric fidelity. Such capability is particularly important for constructing highly interconnected TPMS architectures with complex flow channels, which are difficult to achieve using conventional LPBF systems that typically produce feature sizes of several hundred micrometers. The system is equipped with a 500 W IPG fiber laser and operates in a controlled Argon atmosphere, maintaining oxygen levels below 100 ppm throughout the build process. Key processing parameters included a laser power of 150 W, a scanning speed of 1500 mm/s, and a layer thickness of 20 μm. Benefiting from the rapid solidification characteristics of the μLPBF process, the fabricated Zr-based MG scaffolds exhibit excellent compositional homogeneity without observable elemental segregation.

The geometric characteristics of the three TPMS structures are summarized in Table S1. These TPMS geometries can be mathematically described using implicit equations that approximate their surfaces [1]. The Diamond (D), Gyroid (G) and Fischer-Koch S (S) surfaces are defined by the following approximations:

$$\Phi_D(x,y,z) = \sin(\omega x)\sin(\omega y)\sin(\omega z) + \cos(\omega x)\sin(\omega y)\sin(\omega z) + \sin(\omega x)\cos(\omega y)\sin(\omega z) + \sin(\omega x)\sin(\omega y)\cos(\omega z) = c$$

$$\Phi_G(x,y,z) = \cos(\omega x)\sin(\omega y) + \sin(\omega z)\cos(\omega y) + \sin(\omega x)\cos(\omega z) = c$$

$$\Phi_S(x,y,z) = \cos(2\omega x)\sin(\omega y)\cos(\omega z) + \cos(2\omega y)\sin(\omega z)\cos(\omega x) + \cos(2\omega z)\sin(\omega x)\cos(\omega y) = c \qquad (1)$$

x, y, and z represent the spatial coordinates, while $\omega = 2\pi/l$, where l denotes the length of a unit cell. The process involved extracting the zero-level surface defined by Equation 1, where $\Phi = 0$, and then uniformly offsetting this surface in both directions. Through employing a single-track scanning strategy to achieve the feature size, the thickness under was 100 μm. X-ray diffraction (XRD, Bruker D8 with Cu source, Germany) was then utilized to determine the amorphous structure of the specimens.

Synthesis of NiMo@Zr MMCs

Several chemical reagents were purchased from Macklin (Shanghai, China), including nickel sulfate hexahydrate ($NiSO_4 \cdot 6H_2O$, 98.5%), sodium molybdate dihydrate ($Na_2MoO_4 \cdot 2H_2O$, 99.5%), trisodium citrate dihydrate ($C_6H_5Na_3O_7 \cdot 2H_2O$, 99.5%), sodium chloride (NaCl, 99.5%), and

ammonium hydroxide solution (NH_4OH, 25–28%). All reagents and solvents were used as received from commercial suppliers without further purification.

The 3D printed Zr-based MG substates were carefully detached from the baseplate via wire cutting, then subjected to ultrasonic cleaning to ensure surface integrity before the PED process. Subsequently, NiMo coating was in situ electrochemically deposited on the Zr-based MG structure using an electrolyte solution composed of 0.375 M $NiSO_4 \cdot 6H_2O$, 0.25M $Na_2MoO_4 \cdot 2H_2O$, 0.17M NaCl and 0.55M H_5NO. Electrodeposition was performed in a three-electrode system employing a saturated Hg/HgO reference electrode, the High-Purity Platinum Sheet counter electrode, and the Zr-based MG substrate as the working electrode. Next, the deposition parameters were carefully controlled with pulse on (0.25A, 16 ms) and off (0 mA, 4 ms) for 1 hour at 323 K. After the detailed characterization, both anodic reaction (OER) and cathodic reaction (HER) were conducted in an alkaline solution (1 M KOH) to test the catalytic performance.

Microstructure characterizations and compression tests

Atomic-scale characterization of the NiMo@Zr MMCs was conducted using a scanning electron microscope (SEM, ThermoFisher Verios 5UC). Transmission electron microscopy (TEM) analyses of the NiMo flower-like structures were carried out using a Talos F200X G2 (ThermoFisher) and a JEM-F200 (JEOL) microscope. Mechanical performance was evaluated through uniaxial compression tests using an Instron 68TM-50 Universal Testing Machine (maximum load capacity: 50 kN). All tests were performed at a constant strain rate of 1.0×10^{-3} s^{-1} and were terminated upon complete structural collapse.

3D model reconstruction and Voxel-based finite volume method (FVM)

Geometric deviations introduced during the LPBF process were quantitatively assessed using micro-focus computed tomography (micro-CT, ZEISS Xradia Versa610). The 3D reconstructed models of the printed TPMS microlattices were imported into VGSTUDIO software for fluid permeability and matter transport analysis. Fluid flow simulations were conducted using the Navier–Stokes equations for incompressible fluids with constant density and viscosity. A pressure gradient of 1 Pa was applied across all models, with the outlet pressure fixed at zero to evaluate steady-state flow behavior.

$$\rho \frac{\partial \upsilon}{\partial t} - (\upsilon \cdot \nabla)\upsilon + \frac{\nabla P}{\rho} - \mu \nabla^2 \upsilon - F = 0 \qquad (2)$$

Where ρ is the density of the transport fluid (kg/m^3), υ is the fluid velocity (mm/s), and μ is the dynamic viscosity coefficient of the fluid (Pa s); ∇ and P are the del operator and pressure (Pa), respectively; and F denotes other forces (gravity or centrifugal force; in this case, F = 0). $\upsilon \cdot \nabla = 0$.

Electrochemical measurements

All electrochemical measurements were conducted at 323 K using an electrochemical workstation (CHI 660E, Shanghai, China). The electrocatalytic performance for both the HER and

OER was evaluated in 1.0 M KOH using a standard three-electrode setup, with the synthesized NiMo@Zr MMCs directly serving as the working electrode. Contact angle measurements were performed using a Data Physics OCA15 instrument, with both water droplets and gas bubbles set to a volume of 4 μL. For OER testing, high-purity oxygen was continuously bubbled through the electrolyte to saturate the solution and maintain the O_2/H_2O equilibrium potential at 1.23 V vs. RHE. For HER testing, high-purity nitrogen was purged through the electrolyte for 30 minutes before and during the measurements to remove dissolved oxygen. The polarization curves were obtained by linear sweep voltammetry at a low scan rate of 0.5 mV s^{-1} to eliminate the influence of double capacitance. All polarization curves presented in this work were undertaken iR-corrected with a level of 90%. The Tafel slope was calculated according to the Tafel equation, as follows: $\eta = b \log j + a$, where η, j, b, and a are the potential (V), current density (mA cm^{-2}), Tafel slope (mV dec^{-1}), and intercept, respectively. The chronopotentiometry tests were recorded by applying a current density of 10 mA cm^{-2}. Electrochemical impedance spectroscopy (EIS) was conducted at the open-circuit potential of each sample over a frequency range of 100 kHz to 0.01 Hz, using an AC voltage amplitude of 5.0 mV. Cyclic voltammetry (CV) measurements were performed within a narrow non-Faradaic potential window at varying scan rates (20, 40, 60, 80, and 100 mV s^{-1}) to determine the double-layer capacitance (C_{dl}), which was then used to estimate the electrochemically active surface area (ECSA).

Density functional theory (DFT) calculation

Classical molecular dynamics (MD) simulations were performed using the LAMMPS package. Interatomic interactions were described by a machine-learning spectral neighbor analysis potential (SNAP) specifically developed for Ni–Mo systems and validated against first-principles calculations[2] Two bulk amorphous models with compositions $Ni_{78}Mo_{22}$ and $Ni_{68}Mo_{32}$ were generated, each containing 4000 atoms randomly distributed within periodic simulation cells. The systems were first heated to 2000 K and equilibrated in the NPT ensemble (P = 0) for 1.0 ns to ensure complete melting and compositional homogenization. Subsequently, the liquid configurations were equilibrated at 1500 K for an additional 1.0 ns to obtain well-relaxed liquid structures. To construct the amorphous $Ni_{78}Mo_{22}$/ $Ni_{68}Mo_{32}$ interface, the equilibrated $Ni_{78}Mo_{22}$ liquid cell was divided along the x-direction and a vacuum layer was introduced. The equilibrated $Ni_{68}Mo_{32}$ liquid model was then inserted into the vacant region, generating a composite system containing approximately 8000 atoms. After eliminating atomic overlaps through energy minimization, atoms within 2 nm of the two interfacial regions were allowed to relax while atoms in the bulk regions were temporarily constrained. The interface regions were equilibrated at 1500 K for 0.1 ns to release interfacial stresses and promote structural relaxation across the compositionally distinct amorphous domains. The entire system was subsequently quenched from 1500 K to 300 K, followed by equilibration at 300 K for 0.1 ns and final energy minimization to obtain a stable amorphous–amorphous interfacial structure.

All calculations were carried out using spin-polarized density functional theory (DFT) within the projector augmented wave (PAW) framework, as implemented in the Vienna *Ab Initio* Simulation Package (VASP)[3]. The generalized gradient approximation (GGA) proposed by Perdew, Burke, and Ernzerhof (PBE) was employed for the exchange–correlation potential[4,5]. Long-range van der Waals interactions were accounted for using the DFT-D3 dispersion correction

method[6]. The plane-wave cutoff energy was set to 480 eV. Electronic self-consistency was achieved with an energy convergence threshold of 10^{-5} eV for the iterative solution of the Kohn–Sham equations. All atomic structures were fully relaxed until the residual forces on each atom were less than 0.02 eV/Å. Data analysis and visualization were performed using the VASPKIT[7] package and VESTA[8]. To avoid interlaminar interactions, a vacuum spacing of 20 Å is applied perpendicular to the slab.

Here, differences in Gibbs free energy (ΔG) for intermediates defined as:

$$\Delta G = \Delta E + \Delta E_{ZPE} - T\Delta S + \Delta G_U \quad (3)$$

Here, ΔG represents the total energy difference between the slab and the respective terminations, as calculated using DFT with the PBE functional. ΔEZPE and TΔS denote the differences in zero-point energy and entropy, respectively, between the adsorbed reaction intermediates and the gas phase. T is the temperature, set at 298.15 K. ΔGU = −eU, whereby U is the electrode potential.

Here, we define $\Delta\rho_{A-B} = \Delta\rho_{A+B} - \Delta\rho_A - \Delta\rho_B$ as the charge density difference of A/B heterostructure, where $\rho_{A/B}$, ρ_A and ρ_B are the charge densities of A/B heterostructure, isolated A and B slabs, respectively.

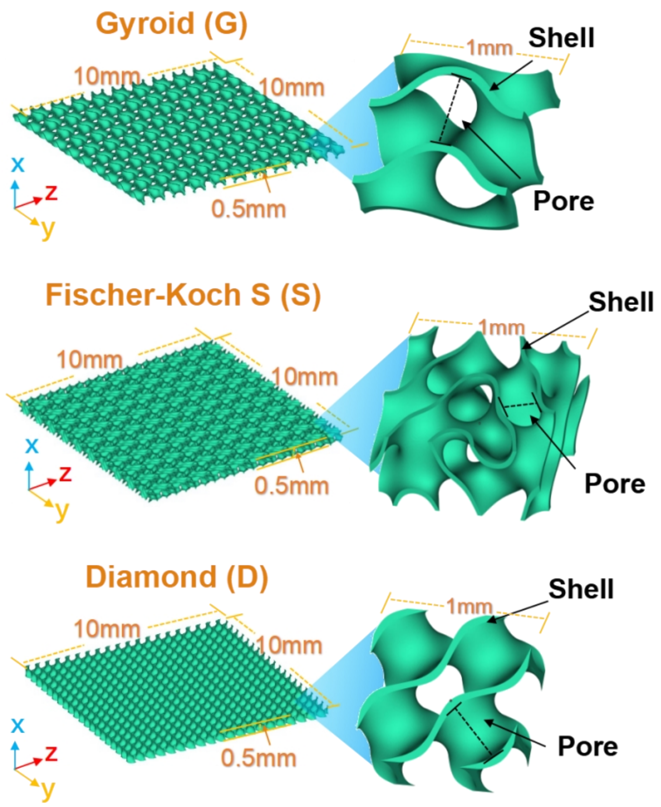

Figure S1. CAD model of three types of TPMS structures of MG scaffold: Fischer-Koch-S (S), Gyroid (G) and Diamond (D).

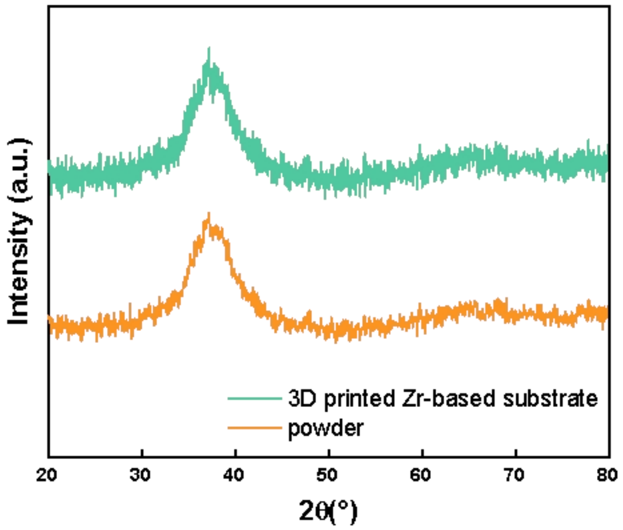

Figure S2. XRD plots of MG powders and 3D printed Zr-based MG substrate.

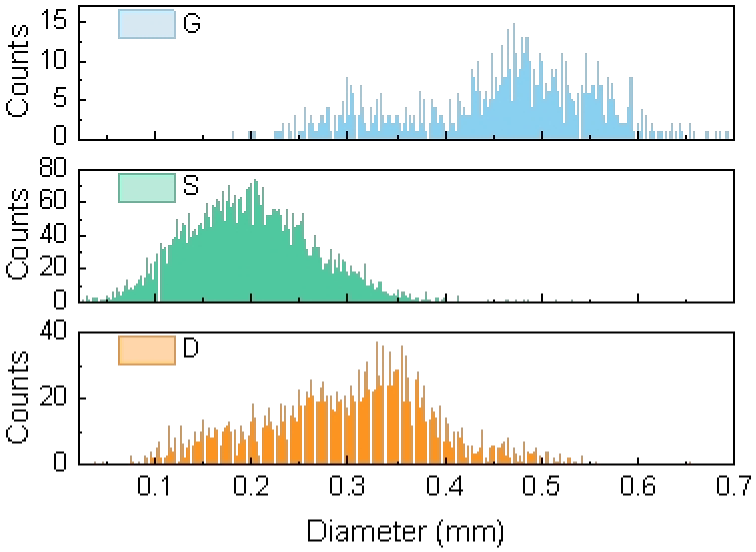

Figure S3. Pore size distribution derived from micro-CT-reconstructed 3D models of three types of TPMS lattices: G, S, D.

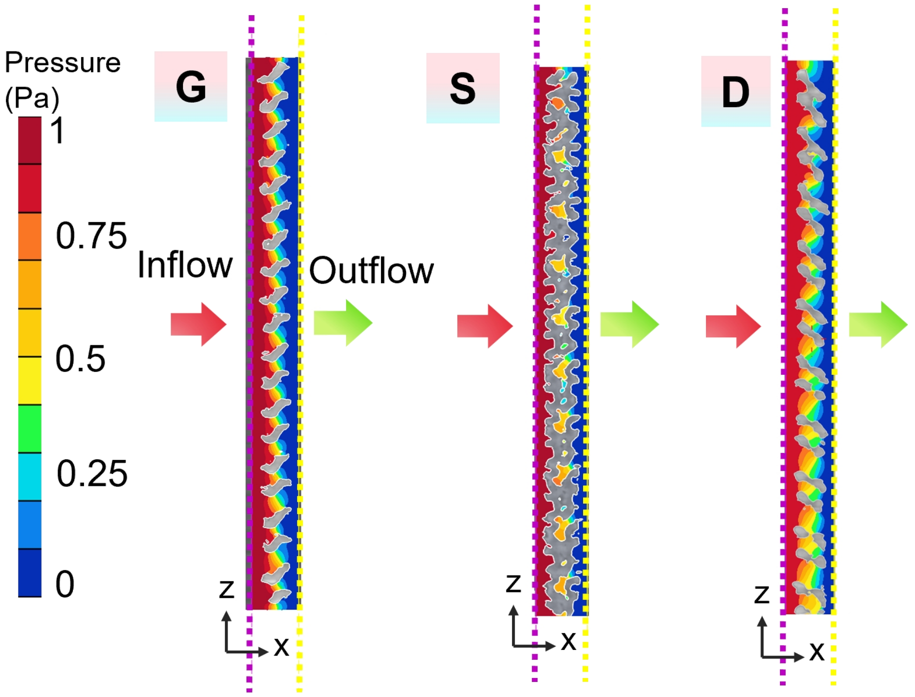

Figure S4. Pressure contours (Pa) for different types of TPMS lattices.

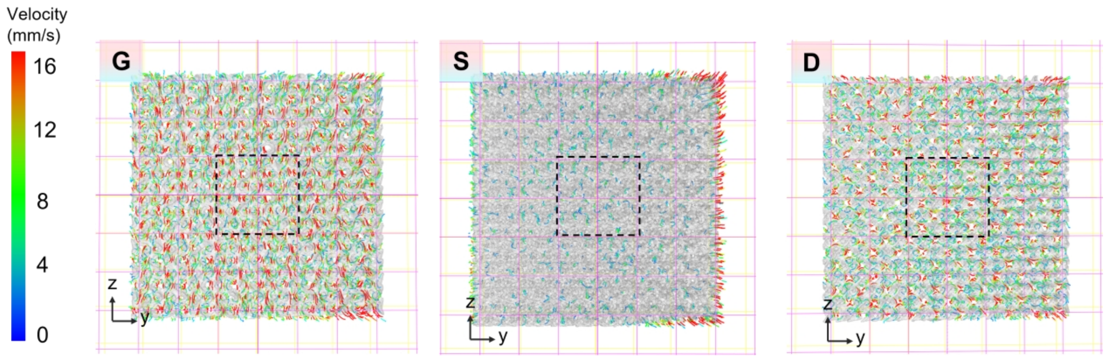

Figure S5. Velocity streamlines for different types of TPMS lattices.

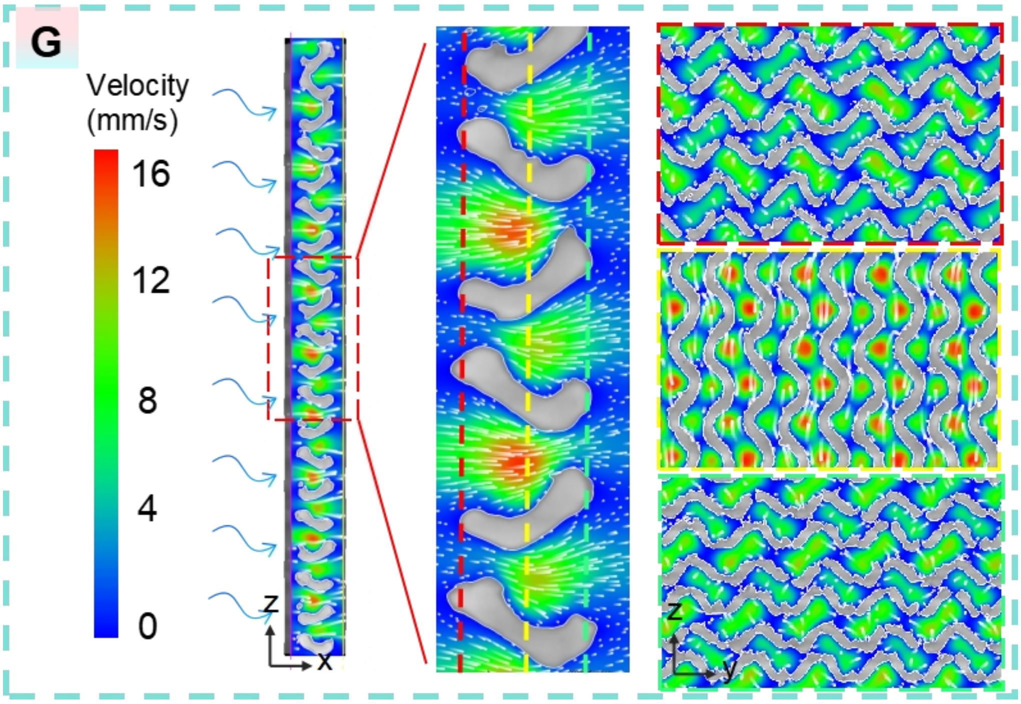

Figure S6. Velocity contours of G-type substrate in y direction and x direction.

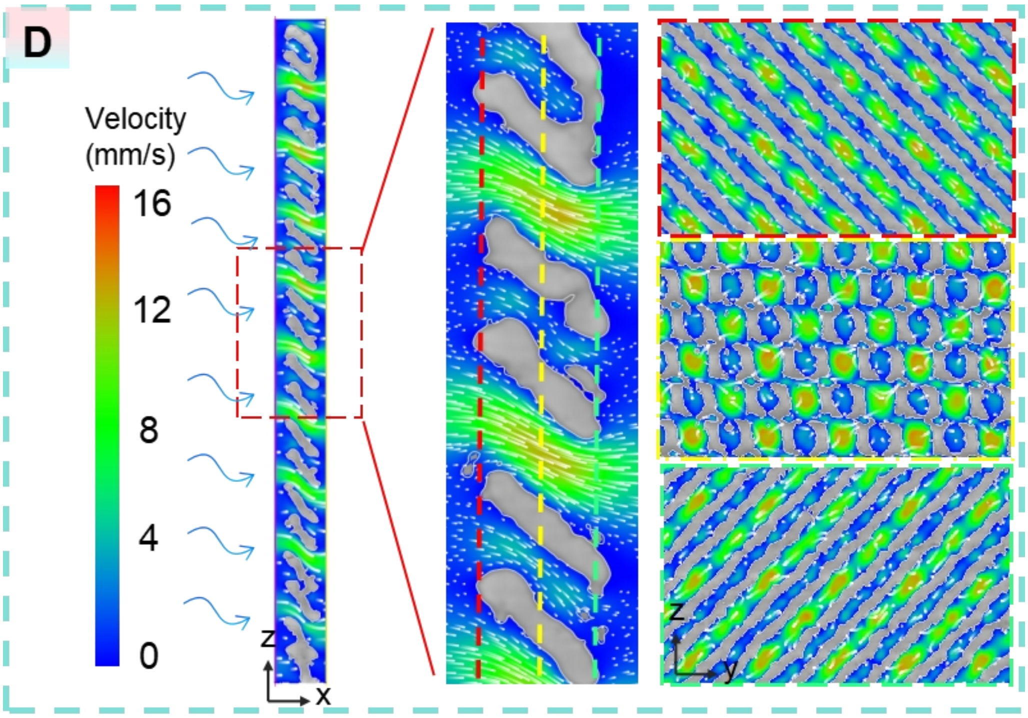

Figure S7. Velocity contours of D-type substrate in y direction and x direction.

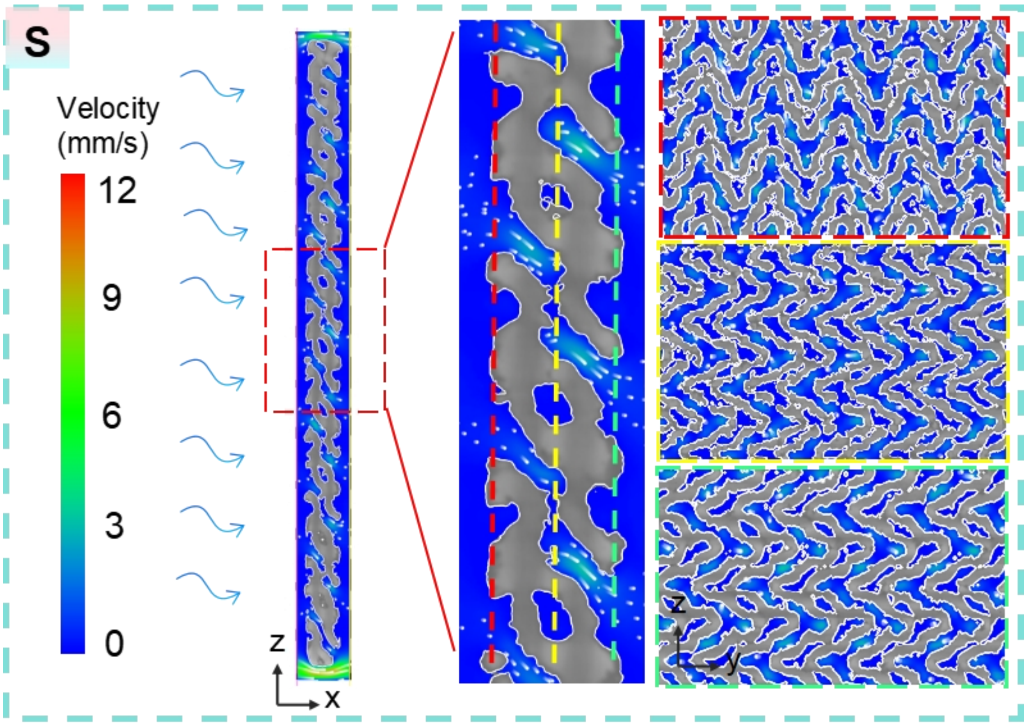

Figure S8. Velocity contours of S-type substrate in y direction and x direction.

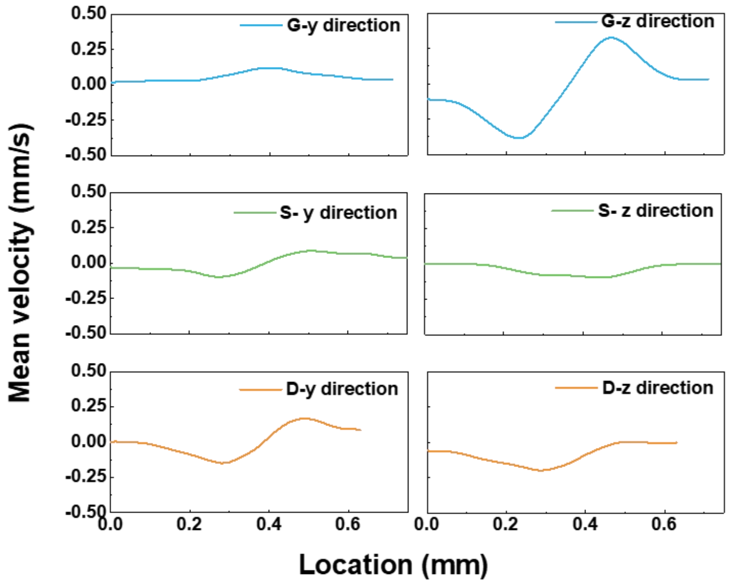

Figure S9. Transverse flow components (y- and z-directions) quantified by mean flow velocity magnitude, highlighting anisotropic transport behavior.

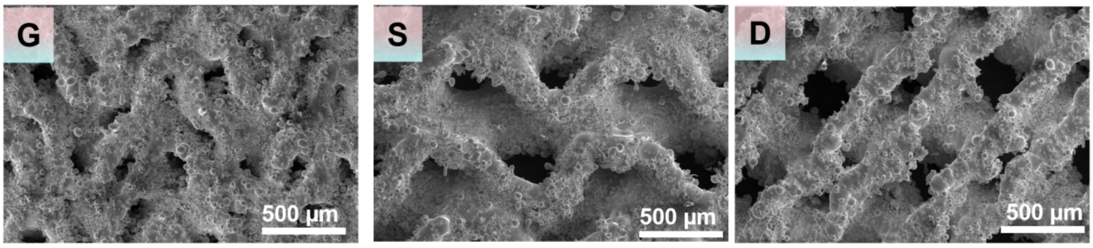

Figure S10. SEM images of as-printed Zr-based MG scaffolds with three different types of TPMS lattices.

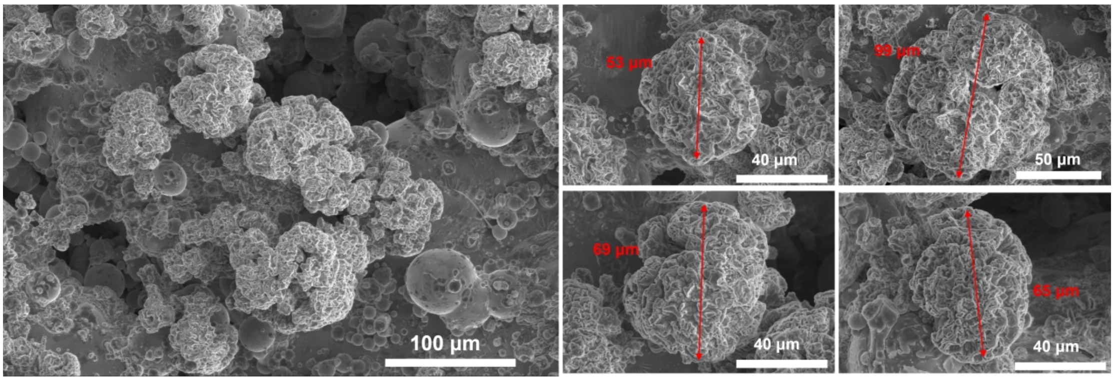

Figure S11. SEM images of hydrangea-shaped NiMo flowers grown on the scaffolds.

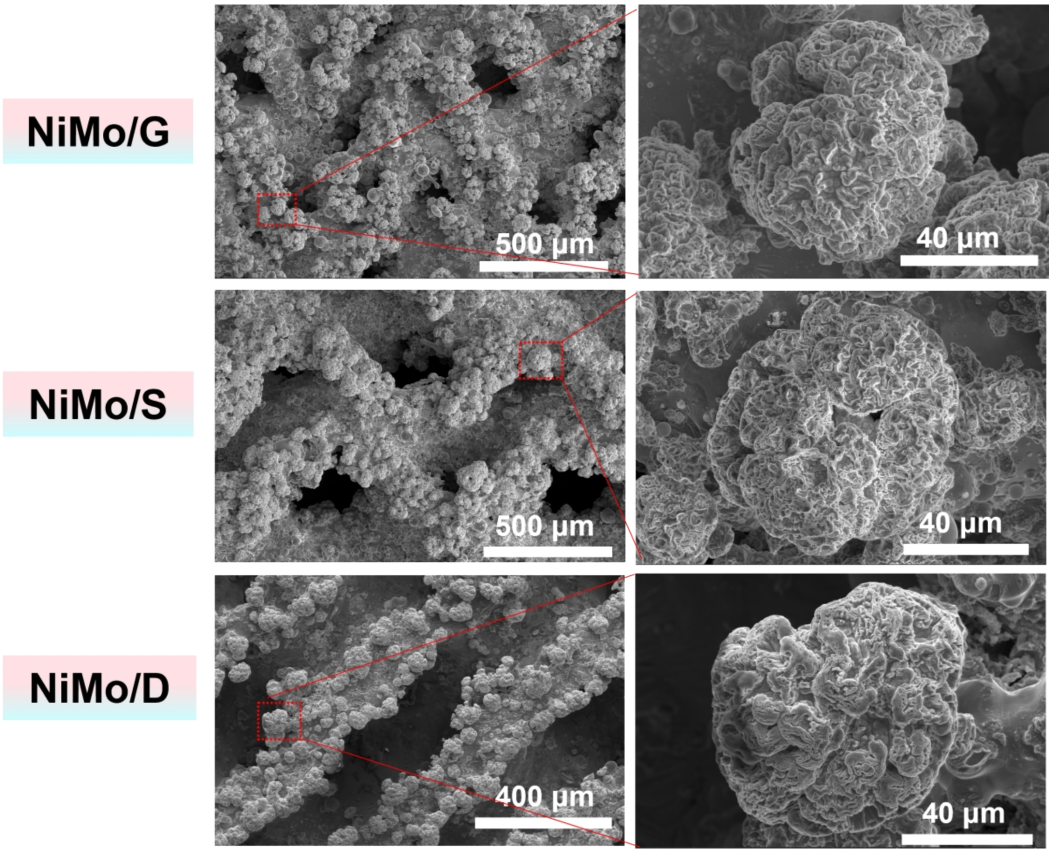

Figure S12. SEM images of hydrangea shaped NiMo flowers coated on the different types of scaffolds.

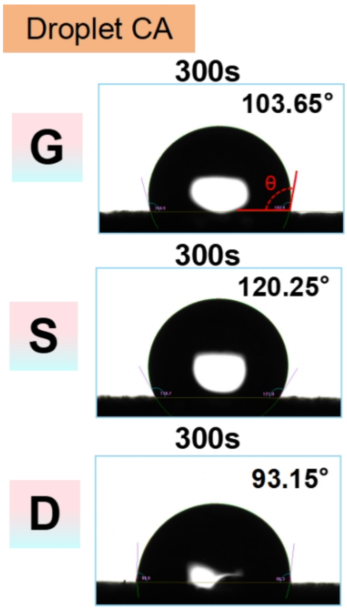

Figure S13. Images of water contact angles of different types of scaffolds.

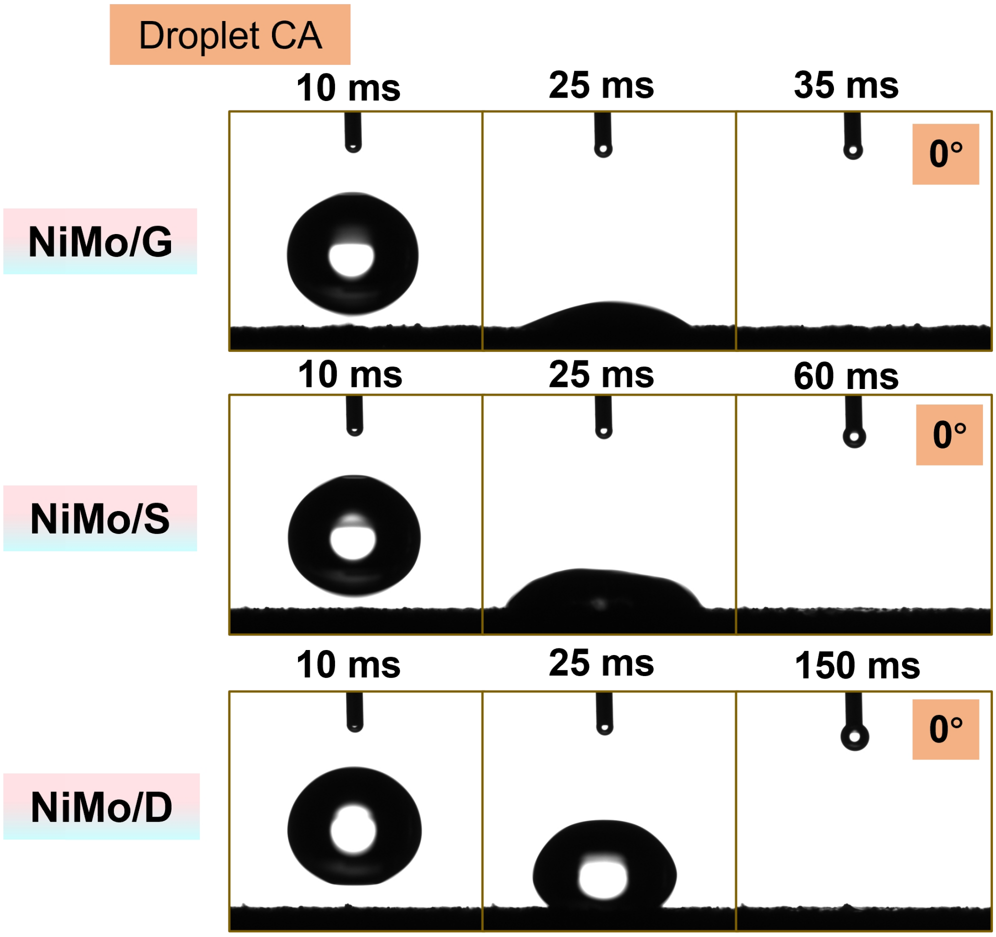

Figure S14. The wetting behavior demonstrating superhydrophilic properties of different types of NiMo@Zr MMCs

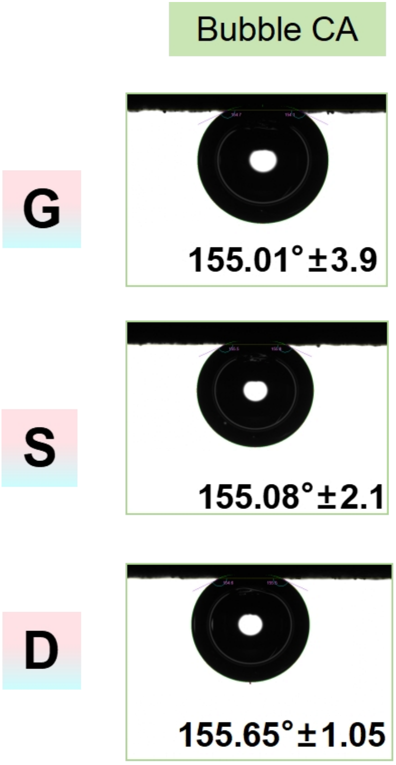

Figure S15. The bubble contact angle tests of different types of scaffolds.

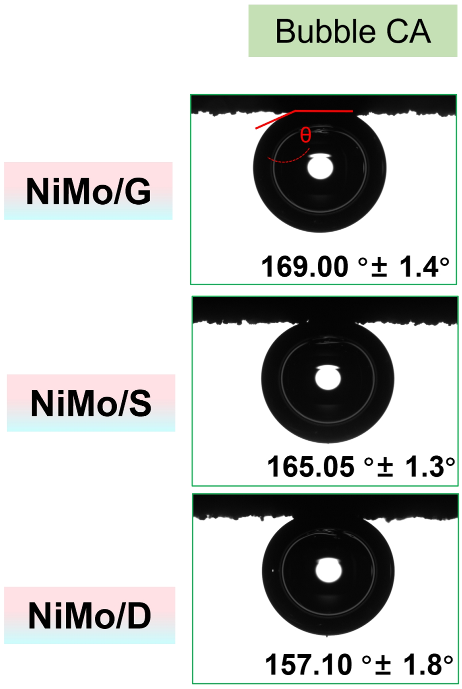

Figure S16. The bubble contact angle tests of different types of NiMo@Zr MMCs.

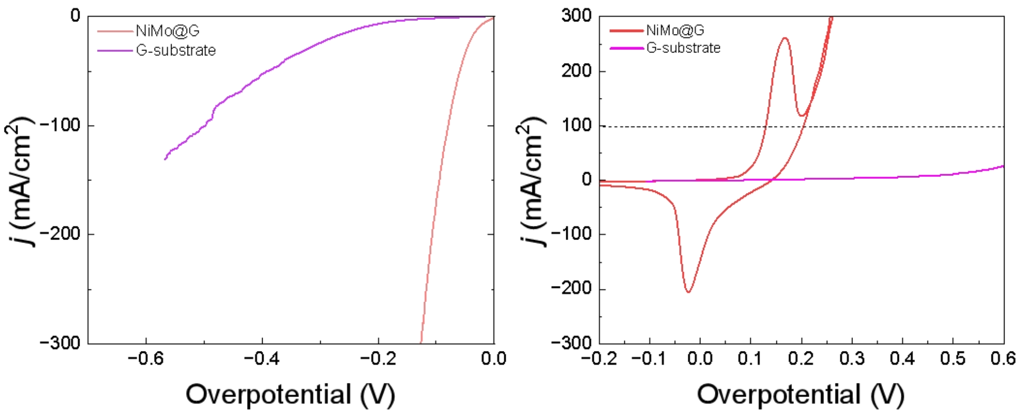

Figure S17. HER and OER polarization curves of G scaffold.

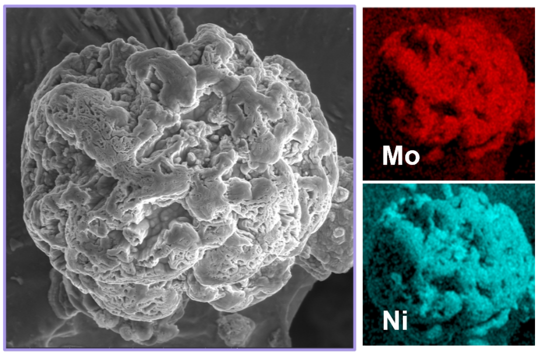

Figure S18. EDX elemental mapping showing the uniform distribution of Ni (cyan) and Mo (red) elements across the deposited structures.

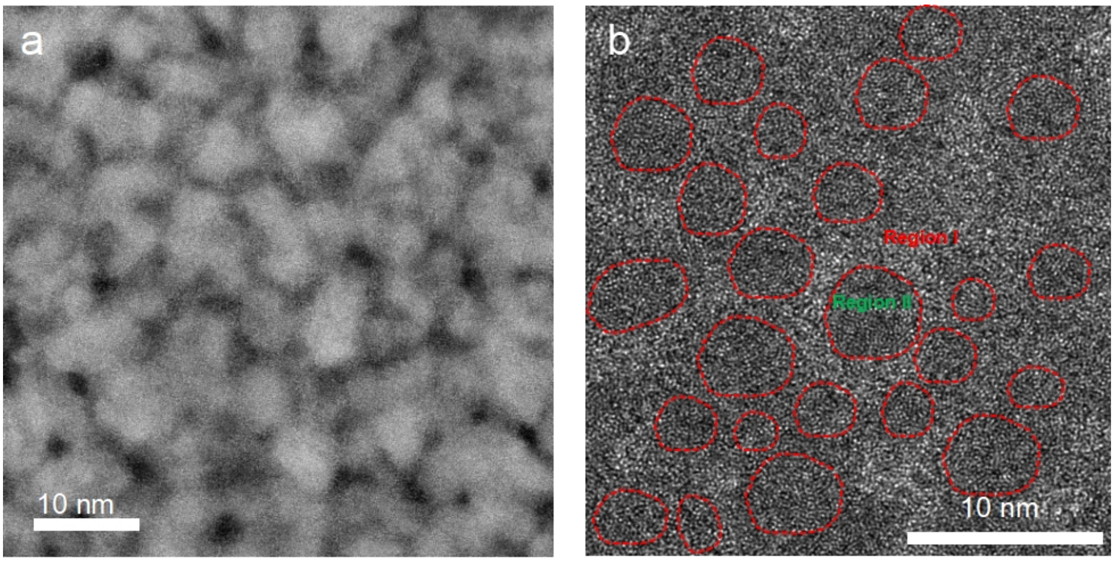

Figure S19. (a) HADDF and (b) HRTEM images of NiMo flower, two distinct regions found in the image: region (I) and region (II).

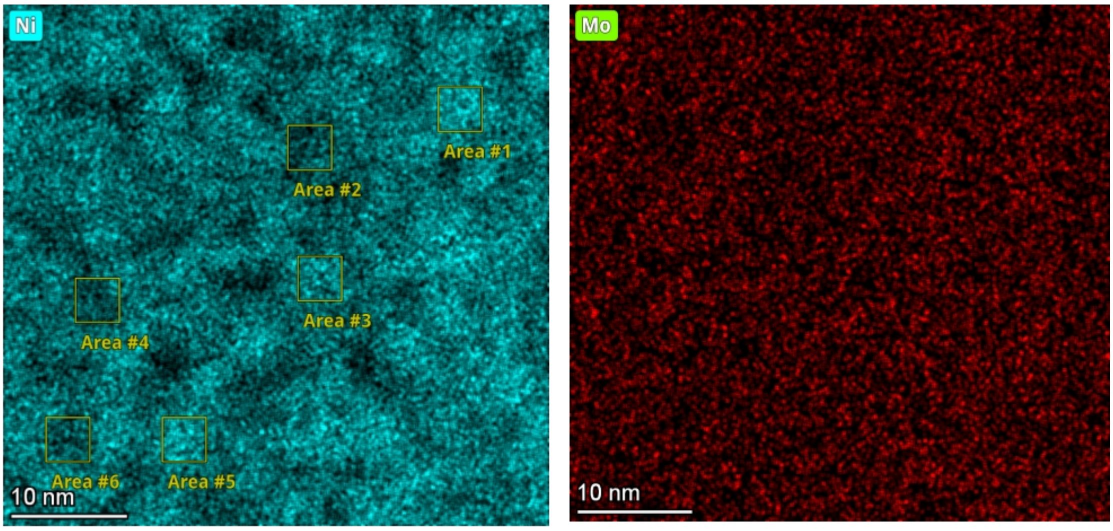

	Area#1	Area#3	Area#5	
Ni(at%)	76.27	79.16	79.6	**78.343**
Mo(at%)	23.73	20.84	20.4	**21.656**

	Area#2	Area#4	Area#6	
Ni(at%)	69.11	64.82	70.17	**68.033**
Mo(at%)	30.89	35.18	29.83	**31.966**

Figure S20. EDX mapping of the Ni and Mo elements

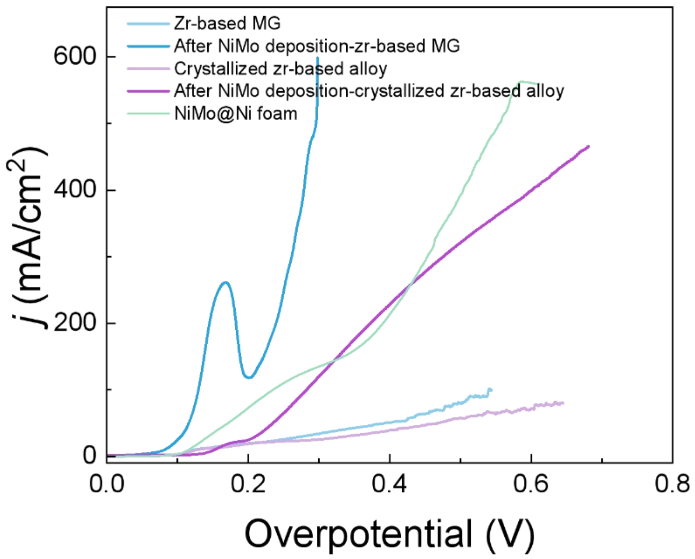

Figure S21. OER performance comparison of NiMo catalysts supported on MG scaffold, crystallized scaffold, and Ni foam substrates.

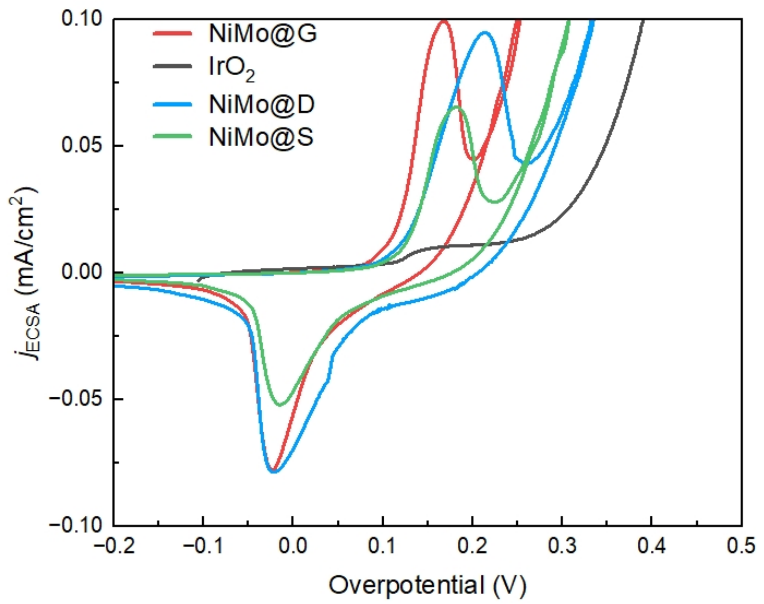

Figure S22. Specific activity (mA/cm2(ECSA)) of NiMo@G, NiMo@S and NiMo@D.

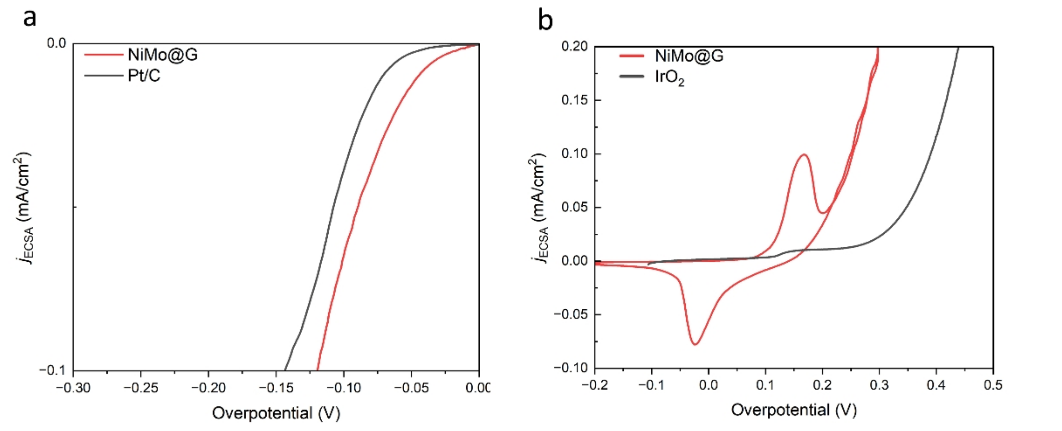

Figure S23. ECSA-normalized HER and OER polarization curves of NiMo@G and commercial catalysts.

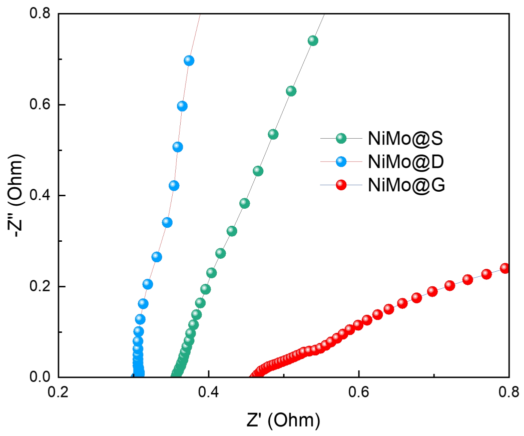

Figure S24. EIS Nyquist plots.

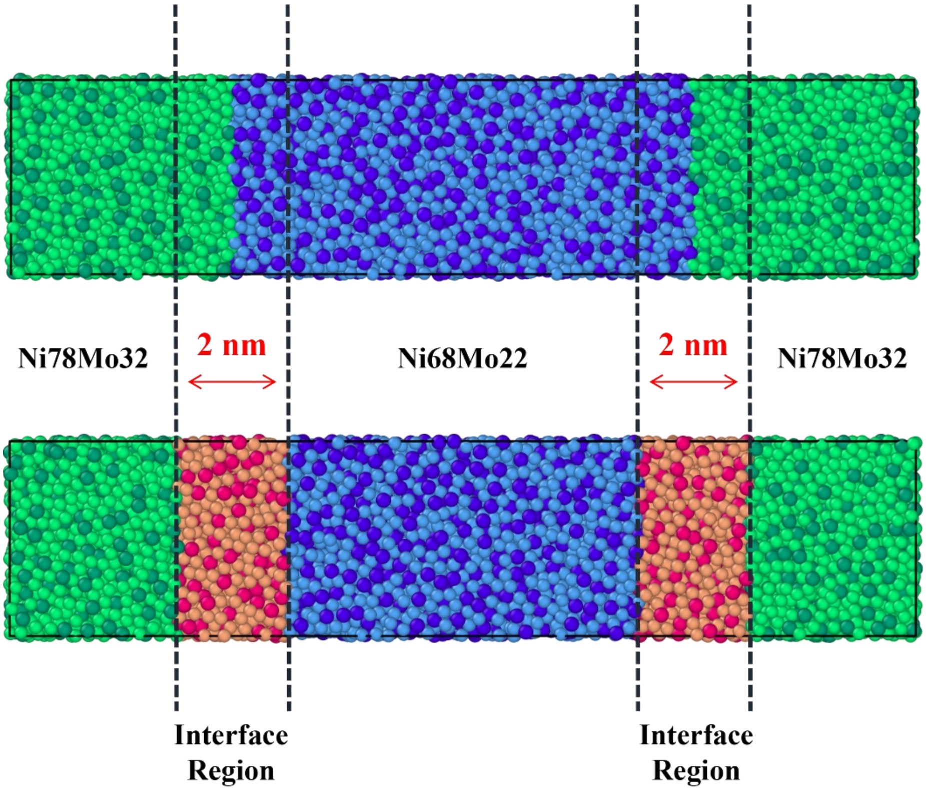

Figure S25. Construction of the amorphous $Ni_{78}Mo_{22}$/$Ni_{68}Mo_{32}$ interfacial model used for statistical DFT calculations.

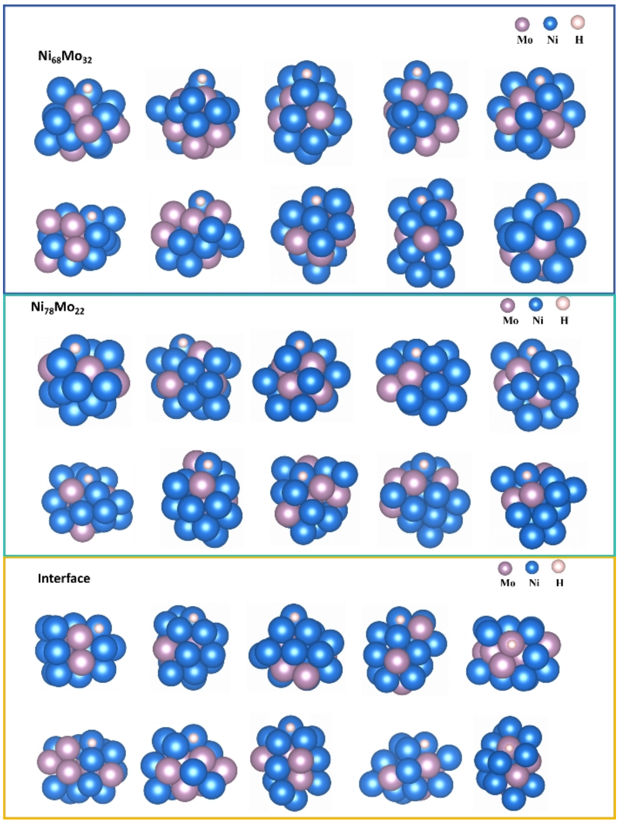

Figure S26. Representative local atomic clusters randomly sampled from the $Ni_{78}Mo_{22}$ phase, $Ni_{68}Mo_{32}$ phase, and amorphous interfacial region for statistical DFT calculations

To account for the intrinsic structural heterogeneity of amorphous materials, ten independent local environments were randomly selected from each of the $Ni_{78}Mo_{22}$ phase, $Ni_{68}Mo_{32}$ phase, and interfacial region. These representative local clusters were subsequently used for first-principles calculations and statistical analysis of hydrogen adsorption behavior

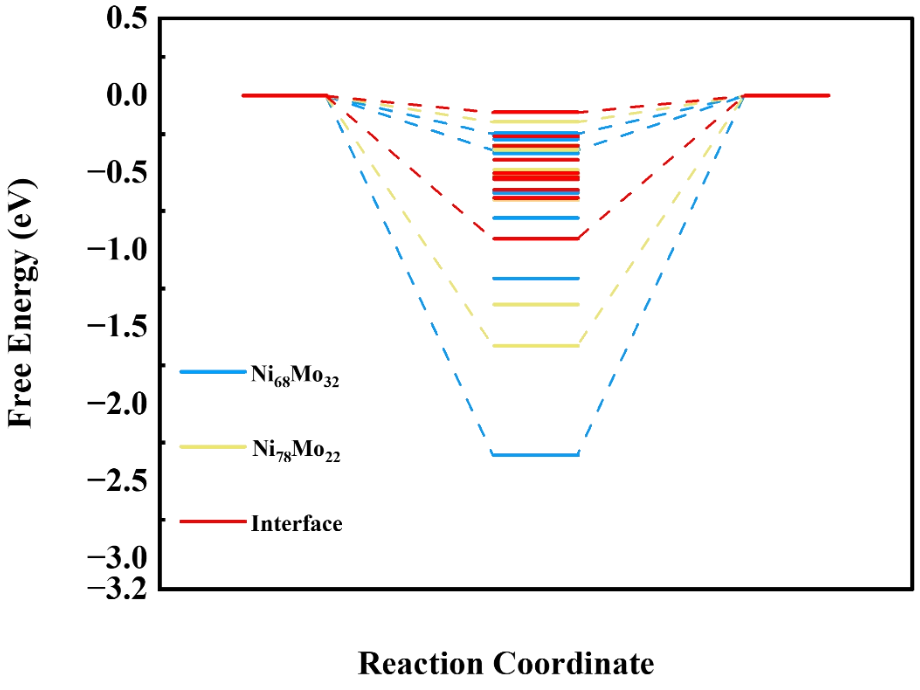

Figure S27. Statistical ΔGH distributions of representative local environments in amorphous NiMo phases and interfaces.

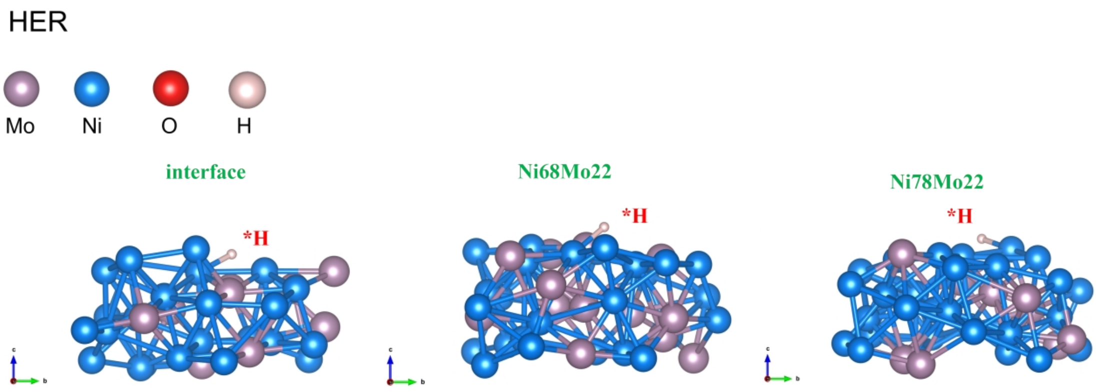

Figure S28. Chemisorption of H on Ni Sites of the different models.

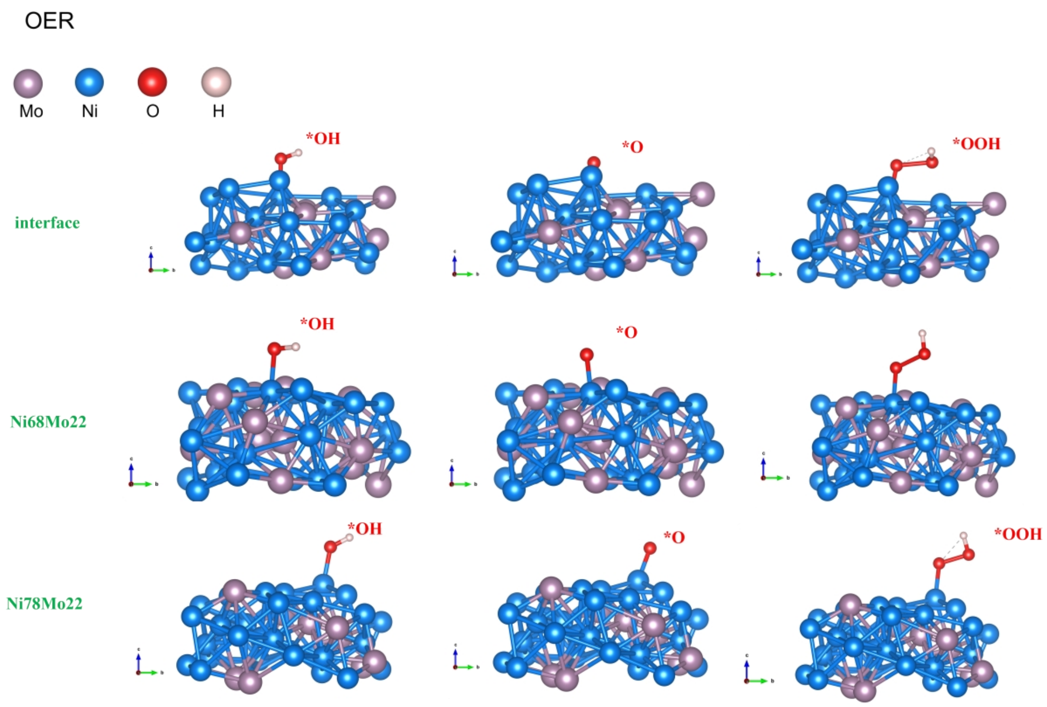

Figure S29. The flow chart of OER at Ni sites of the different models.

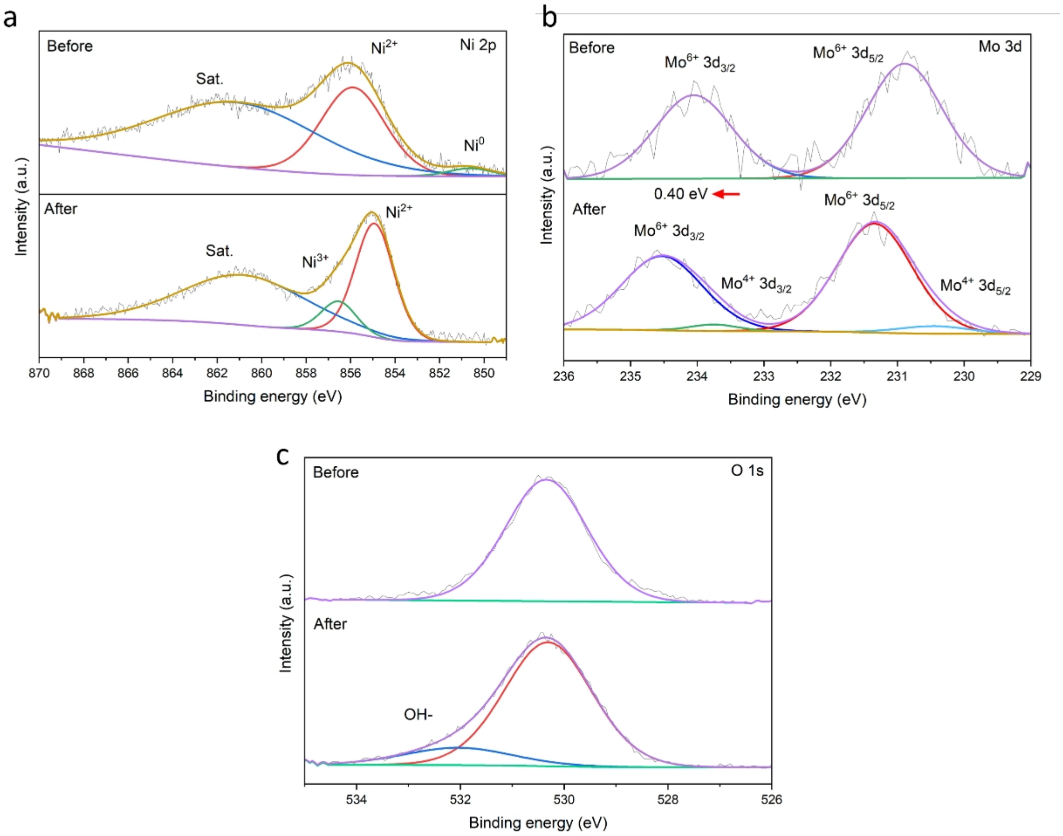

Figure S30. XPS survey spectra of NiMo@G before and after the stability test.

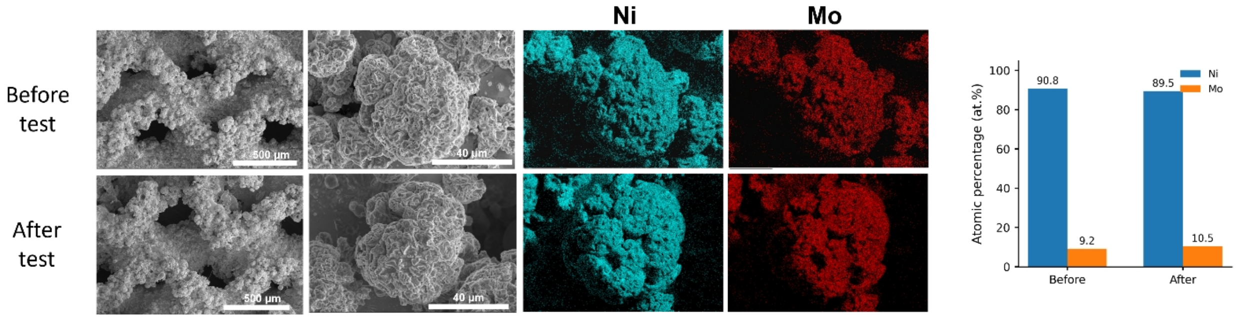

Figure S31. SEM images and EDX mapping of NiMo@G before and after the 200h stability test.

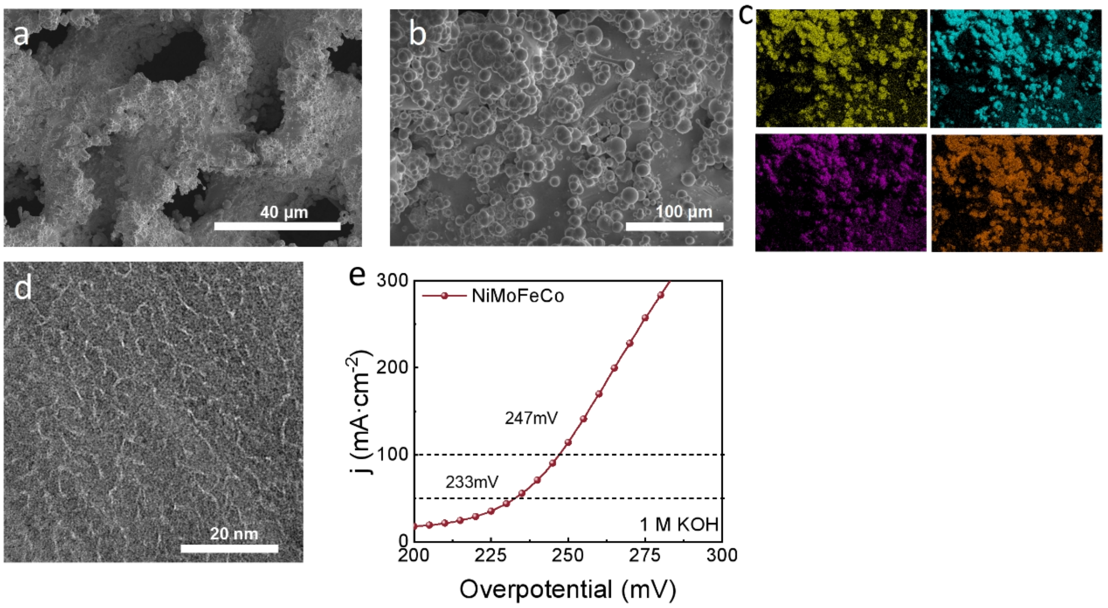

Figure S32. Preliminary demonstration of the compositional adaptability of the proposed multiscale metamaterial catalyst framework. (a) Low-magnification SEM image of the NiMoFeCo coating deposited on the Gyroid scaffold. (b) High-magnification SEM image showing the evolved surface morphology. (c) EDX elemental mapping of Ni, Mo, Fe, and Co. (d) TEM image revealing the amorphous/nanocrystalline heterogeneous structure. (e) OER polarization curve of the NiMoFeCo@ G catalyst.

Table S1 Geometrical characteristics of the 3D printed Zr-based MG lattice scaffolds.

**Lattice sample code**	**Unit cell size(mm)**	**Number of cells(x,y,z)**	**Measured average *m*(g)**	**Measured porosity (%) from CT**
S	1	10*10*0.5	0.244±0.02	52.0%
G	1	10*10*0.5	0.135±0.02	72.3%
D	1	10*10*0.5	0.127±0.03	71.7%

Table S2 Comparison of the overpotentials of NiMo@G catalyst and recently reported noble metal catalyst for overall water splitting at 100 mA cm-2 in 1.0 M KOH solution.

Electrocatalysts	*Current density (mA cm^{-2})*	*Cell Voltage (V)*	*References*
NiMo@G	100	1.438	This work
NiFeMoS/NF-P	100	1.52	[9]
Ni-Co-Fe-P NBs	100	1.58	[10]
a/c-RuCoMoyOx/NF	100	1.578	[11]
Ru-CoOx/NF	100	1.59	[12]
$NiMoO_x$/NiMoS	100	1.63	[13]
Mo-NiPx/NiSy	100	1.7	[14]
Ni-MoO2/NF-IH	100	1.75	[15]
NW-MnCo2O4/GDY	100	1.76	[16]
Ni-Ru-Fe@CNT	100	1.77	[17]
Pt/C//IrO2	100	1.783	This work
CuNi@NiFeCu	100	1.8	[18]
Pt@CoS_2-NrGO	100	1.84	[19]
CoMoS-NF-31	100	2.1	[20]

Table S3 General characteristics comparison of the common materials used for overall water splitting.

Types	Materials	Mass Transfer	Hydrophilic/Hydrophobic	Mechanical strength	Economics cost	Catalytic performance	References
Non-porous precious metal catalysts	Pt-Ni_2Fe_1-T	√	√ √ √	√ √ √	√	√ √ √ √ √	21
Porous Metal catalysts	NiFe-MOF	√	√ √ √ √	√	√ √ √ √ √	√ √ √ √	22
	Cu2O/Cu	√	√ √ √ √	√ √ √	√ √ √ √ √	√ √ √ √	23
	N-(Co-Cu) Sx	√	√ √ √ √	√ √	√ √ √ √ √	√ √ √ √	24
3D printed metal microlattices	316 L	√ √ √ √ √	√ √ √ √	√ √ √ √	√ √ √ √ √	√	25
This work	*NiMo@G*	√ √ √ √ √	√ √ √ √ √	√ √ √ √ √	√ √ √ √ √	√ √ √ √ √	

References

1 Rajagopalan, S. & Robb, R. A. Schwarz meets Schwann: Design and fabrication of biomorphic and durataxic tissue engineering scaffolds. *Medical Image Analysis* **10**, 693-712, doi:https://doi.org/10.1016/j.media.2006.06.001 (2006).

2 Li, X.-G. *et al.* Quantum-accurate spectral neighbor analysis potential models for Ni-Mo binary alloys and fcc metals. *Physical Review B* **98**, 094104, doi:10.1103/PhysRevB.98.094104 (2018).

3 Kresse, G. F. l., J. Efficient iterative schemes for ab initio total-energy calculations using a plane-wave basis set. *Physical Review B - Condensed Matter* **54**, 169-185, doi:https://doi.org/10.1103/PhysRevB.54.11169 (1996).

4 John P. Perdew, K. B., and Matthias Ernzerhof. Generalized gradient approximation made simple. *Phys. Rev. Lett* **77**, doi:https://doi.org/10.1103/PhysRevLett.77.3865 (1996).

5 Kohn, W. & Sham, L. J. Self-Consistent Equations Including Exchange and Correlation Effects. *Physical Review* **140**, A1133-A1138, doi:https://doi.org/10.1103/PhysRev.140.A1133 (1965).

6 Grimme, S., Antony, J., Ehrlich, S. & Krieg, H. A consistent and accurate ab initio parametrization of density functional dispersion correction (DFT-D) for the 94 elements H-Pu. *The Journal of chemical physics* **132**, 154104, doi:https://doi.org/10.1063/1.3382344 (2010).

7 Wang, V., Xu, N., Liu, J.-C., Tang, G. & Geng, W.-T. VASPKIT: A user-friendly interface facilitating high-throughput computing and analysis using VASP code. *Computer Physics Communications* **267**, 108033, doi:https://doi.org/10.1016/j.cpc.2021.108033 (2021).

8 Momma, K. & Izumi, F. VESTA: a three-dimensional visualization system for electronic and structural analysis. *Journal of Applied Crystallography* **41**, 653-658, doi:https://doi.org/10.1107/s0021889808012016 (2008).

9 Yan, K.-L. *et al.* Organic-inorganic hybrids-directed ternary NiFeMoS anemone-like nanorods with scaly surface supported on nickel foam for efficient overall water splitting. *Chemical Engineering Journal* **334**, 922-931, doi:https://doi.org/10.1016/j.cej.2017.10.074 (2018).

10 Li, A. *et al.* Rational design of porous Ni-Co-Fe ternary metal phosphides nanobricks as bifunctional electrocatalysts for efficient overall water splitting. *Applied Catalysis B: Environmental* **310**, 121353 (2022).

11 Ren, J. *et al.* Modulating amorphous/crystalline heterogeneous interface in RuCoMoyOx grown on nickel foam to achieve efficient overall water splitting. *Chemical Engineering Journal* **469**, 143993, doi:https://doi.org/10.1016/j.cej.2023.143993 (2023).

12 Wu, D., Chen, D., Zhu, J. & Mu, S. Ultralow Ru incorporated amorphous cobalt-based oxides for high-current-density overall water splitting in alkaline and seawater media. *Small* **17**, 2102777 (2021).

13 Zhai, P. *et al.* Engineering active sites on hierarchical transition bimetal oxides/sulfides heterostructure array enabling robust overall water splitting. *Nature Communications* **11**, 5462, doi:10.1038/s41467-020-19214-w (2020).

14 Wang, J. *et al.* Heterogeneous Bimetallic Mo-NiPx/NiSy as a Highly Efficient Electrocatalyst for Robust Overall Water Splitting. *Advanced Functional Materials* **31**, doi:10.1002/adfm.202101532 (2021).

15 Xiong, G. *et al.* Rapid synthesis of various electrocatalysts on Ni foam using a universal and facile induction heating method for efficient water splitting. *Advanced Functional Materials* **31**, 2009580 (2021).

16 Qi, L. *et al.* 1D nanowire heterojunction electrocatalysts of MnCo2O4/GDY for efficient overall water splitting. *Advanced Functional Materials* **32**, 2107179 (2022).

17 Gao, T. *et al.* Ultra-fast preparing carbon nanotube-supported trimetallic Ni, Ru, Fe heterostructures as robust bifunctional electrocatalysts for overall water splitting. *Chemical Engineering Journal* **424**, 130416 (2021).

18 Cao, D., Xu, H. & Cheng, D. Branch-leaf-shaped CuNi@ NiFeCu nanodendrites as highly efficient electrocatalysts for overall water splitting. *Applied Catalysis B: Environmental* **298**, 120600 (2021).

19 Logeshwaran, N. *et al.* An efficient and durable trifunctional electrocatalyst for zinc–air batteries driven overall water splitting. *Applied Catalysis B: Environmental* **297**, 120405 (2021).

20 Yang, Y. *et al.* Hierarchical Nanoassembly of MoS2/Co9S8/Ni3S2/Ni as a Highly Efficient Electrocatalyst for Overall Water Splitting in a Wide pH Range. *Journal of the American Chemical Society* **141**, 10417-10430, doi:10.1021/jacs.9b04492 (2019).

21 Wang, L. *et al.* In Situ Anchoring Massive Isolated Pt Atoms at Cationic Vacancies of α-NixFe1-x(OH)2 to Regulate the Electronic Structure for Overall Water Splitting. *Advanced Functional Materials* **32**, 2203342, doi:https://doi.org/10.1002/adfm.202203342 (2022).

22 Li, C. *et al.* Interfacial Electronic Interactions Between Ultrathin NiFe-MOF Nanosheets and Ir Nanoparticles Heterojunctions Leading to Efficient Overall Water Splitting. *Advanced Science* **11**, 2401780, doi:https://doi.org/10.1002/advs.202401780 (2024).

23 Zhou, J. *et al.* Achieving efficient and stable electrochemical nitrate removal by in-situ reconstruction of Cu2O/Cu electroactive nanocatalysts on Cu foam. *Applied Catalysis B: Environmental* **317**, 121811, doi:https://doi.org/10.1016/j.apcatb.2022.121811 (2022).

24 He, J.-C. *et al.* Three-dimensional amorphous N-doped cobalt–copper sulfide nanostructures for efficient full water splitting. *Rare Metals*, doi:10.1007/s12598-024-03035-7 (2025).

25 Pham, M. S., Liu, C., Todd, I. & Lertthanasarn, J. Damage-tolerant architected materials inspired by crystal microstructure. *Nature* **565**, 305-311, doi:10.1038/s41586-018-0850-3 (2019).
